# Clinician approaches to spinal manipulation for persistent spinal pain after lumbar surgery: systematic review and meta-analysis of individual patient data

**DOI:** 10.1186/s12998-023-00481-5

**Published:** 2023-03-09

**Authors:** Robert J. Trager, Clinton J. Daniels, Kevin W. Meyer, Amber C. Stout, Jeffery A. Dusek

**Affiliations:** 1 Connor Whole Health, University Hospitals Cleveland Medical Center, 11100 Euclid Ave, Cleveland, OH 44106 USA; 2 College of Chiropractic, Logan University, Chesterfield, MO 63017 USA; 3 VA Puget Sound Health Care System, Rehabilitation Care Services, 9600 Veterans Drive, Tacoma, WA 98493 USA; 4 Lakeside Hospital Library, Cleveland Medical Center, 11000 Euclid Ave, Cleveland, OH 44106 USA; 5 Department of Family Medicine and Community Health, School of Medicine, Case Western Reserve University, Cleveland, OH 44106 USA

**Keywords:** Spinal manipulation, Chiropractic, Lumbosacral region, Clinical decision making, Systematic review, Surgical procedure, Failed back surgery syndrome

## Abstract

**Background:**

This review aimed to identify variables influencing clinicians’ application of spinal manipulative therapy (SMT) for persistent spine pain after lumbar surgery (PSPS-2). We hypothesized markers of reduced clinical/surgical complexity would be associated with greater odds of applying SMT to the lumbar region, use of manual-thrust lumbar SMT, and SMT within 1-year post-surgery as primary outcomes; and chiropractors would have increased odds of using lumbar manual-thrust-SMT compared to other practitioners.

**Methods:**

Per our published protocol, observational studies describing adults receiving SMT for PSPS-2 were included. PubMed, Web of Science, Scopus, OVID, PEDro, and Index to Chiropractic Literature were searched from inception to January 6, 2022. Individual patient data (IPD) were requested from contact authors when needed for selection criteria. Data extraction and a customized risk-of-bias rubric were completed in duplicate. Odds ratios (ORs) for primary outcomes were calculated using binary logistic regressions, with covariates including age, sex, symptom distribution, provider, motion segments, spinal implant, and surgery-to-SMT interval.

**Results:**

71 articles were included describing 103 patients (mean age 52 ± 15, 55% male). The most common surgeries were laminectomy (40%), fusion (34%), and discectomy (29%). Lumbar SMT was used in 85% of patients; and of these patients was non-manual-thrust in 59%, manual-thrust in 33%, and unclear in 8%. Clinicians were most often chiropractors (68%). SMT was used > 1-year post-surgery in 66% of cases. While no primary outcomes reached significance, non-reduced motion segments approached significance for predicting use of lumbar-manual-thrust SMT (OR 9.07 [0.97–84.64], *P* = 0.053). Chiropractors were significantly more likely to use lumbar-manual-thrust SMT (OR 32.26 [3.17–327.98], *P* = 0.003). A sensitivity analysis omitting high risk-of-bias cases (missing ≥ 25% IPD) revealed similar results.

**Conclusions:**

Clinicians using SMT for PSPS-2 most often apply non-manual-thrust SMT to the lumbar spine, while chiropractors are more likely to use lumbar-manual-thrust SMT relative to other providers. As non-manual-thrust SMT may be gentler, the proclivity towards this technique suggests providers are cautious when applying SMT after lumbar surgery. Unmeasured variables such as patient or clinician preferences, or limited sample size may have influenced our findings. Large observational studies and/or international surveys are needed for an improved understanding of SMT use for PSPS-2.

*Systematic review registration* PROSPERO (CRD42021250039).

**Supplementary Information:**

The online version contains supplementary material available at 10.1186/s12998-023-00481-5.

## Introduction

### Rationale

Persistent spinal pain syndrome type 2 (PSPS-2) describes recurrent or chronic axial or radicular spinal pain in patients with a history of spinal surgery, and replaces previous terms describing this clinical presentation such as “failed back surgery syndrome” and post-laminectomy syndrome [1]. Between 20 and 40% of patients develop PSPS-2 after lumbar spine surgery for a variety of reasons including factors present before surgery or progression of degenerative changes [2]. Conservative treatments are typically preferred for PSPS-2, including exercise, medications, epidural injections, and neuromodulation, with revision surgery being reserved for refractory cases [2, 3]. Although spinal manipulative therapy (SMT) is recommended by several clinical practice guidelines for treatment of low back pain [4–6], research is limited regarding its use for those with PSPS-2 [7–10].

Spinal manipulative therapy is defined as any manual therapy technique targeting the spinal joints or vertebrae, excluding soft tissue treatments [11]. SMT is used globally by several disciplines including chiropractors, physical therapists, osteopaths, and traditional East Asian medicine (TEAM) providers [11]. Broadly, SMT can be applied using thrust or non-thrust techniques [12, 13], with thrust-SMT involving a high-velocity, low-amplitude impulse, and non-thrust SMT involving low velocity oscillations [11, 13, 14].

There are a range of reasons why practitioners could justify using SMT for patients with PSPS-2. For those with impaired mobility, SMT might be applied to improve segmental or regional mobility [9]. SMT also produces a hypoalgesic effect [15] which may occur even when SMT is applied at a site away from the source of symptoms [16]. Further, surveys have reported that patients often seek chiropractic care to avoid surgery [17] or taking prescription medications [18], thus it is possible providers may administer SMT in hopes to provide patients with an alternative to pharmacologic treatment or revision surgery.

Precautions to SMT in PSPS-2 have been proposed from narrative reviews on the topic. One review hypothesized that SMT could cause lead migration or failure of implanted spinal cord stimulators (SCS) [9]. In another review, spinal instability or instrumentation dysfunction in patients with lumbar fusion were suggested as considerations prior to SMT use [19]. Despite these potential concerns, a recent systematic review identified no serious adverse events related to SMT use in those with PSPS-2 [8].

Based on limited evidence, we suspected that clinicians’ approach to SMT in patients with PSPS-2 would depend on the complexity of the patient’s surgery. Generally, single-level surgeries without instrumentation such as laminectomies or discectomies are considered less complex and do not affect the number of lumbar motion segments [20, 21]. Conversely, multi-level fusions with instrumentation are more complex and reduce the number of available motion segments [20, 21]. In one study, the majority of a small panel of experts (n = 9) rated both non-thrust and thrust-SMT as “appropriate” for patients with previous laminectomy [22]. In contrast, in a recent survey, over half of chiropractors reported either “rarely” or “never” using thrust-SMT in patients with previous lumbar fusion [10].

Although SMT is used for patients with PSPS-2, there is little evidence to guide which specific SMT techniques are appropriate given the potential precautions in such patients. Given this gap in the literature, this systematic review aims to identify clinical and surgical variables that predict altered SMT technique by synthesizing individual patient data (IPD).

### Objectives

1. Identify variables that predict clinicians’ use of lumbar-SMT, lumbar manual-thrust-SMT, and timing of SMT within 1-year post-surgery in adults with PSPS-2. As a primary outcome, we hypothesize that markers of reduced clinical/surgical complexity including younger age, non-radiating symptoms, no spinal implant(s), and a greater number of motion segments will each be independently associated with greater odds of: (1) lumbar-SMT, (2) lumbar manual-thrust-SMT, and (3) SMT within 1-year post-surgery. As a secondary outcome, we hypothesize that chiropractors will have increased odds of using lumbar manual-thrust-SMT relative to other disciplines.
2. Describe features of adults with PSPS-2 receiving SMT: age, symptom distribution, surgery type, number of lumbar motion segments, spinal implants, post-surgical imaging, SMT type, interval between surgery and SMT, and SMT practitioner type.

## Methods

### Protocol and registration

The review protocol was registered with PROSPERO (CRD42021250039) and was previously published [23]. For additional details regarding the methodology of the current review, please refer to those documents. Reporting of this review was structured according to the Preferred Reporting Items for Systematic Review and Meta-Analysis 2020 (PRISMA2020) and PRISMA-IPD statements [24]. This review was deemed Not Human Subjects by the University Hospitals Institutional Review Board (Cleveland, OH, USA, STUDY20210555).

One deviation from the original protocol was a simplification of the regression models. This was needed as there were few events per category for the variables of motion segments and provider type. As an insufficient number of cases per category is known to produce unreliable estimates [25], these categories were simplified to include a reduced or non-reduced number of motion segments, and treatment by a chiropractor or non-chiropractor, respectively. The number of motion segments was then also classified as a nominal, rather than ordinal variable. This improved our ability to draw inferences from the regression model yet remained consistent with our a priori hypotheses. These simplifications were carried forward into the sensitivity analysis while the original data extraction categories remained as-is for the descriptive synthesis. A final modification consistent with previous guidance for troubleshooting wide and infinite confidence intervals that arose in our initial regression models was to lower the threshold for bivariate correlation, using a Pearson correlation coefficient cutoff of 0.55 rather than 0.70 [26–28]. This allowed us to exclude a predictor variable and ultimately stabilized the regression models, avoiding infinite confidence intervals.

In a minor addition to original protocol, investigators provided free-text description of the SMT technique used in each case. This helped corroborate each investigators’ decision to classify the other SMT data items and was used to supplement the qualitative and quantitative synthesis and discussion. Other deviations included our use of Rayyan [29] instead of Covidence (Veritas Health Innovation Ltd, Australia) for article screening, and use of the statistical software IBM SPSS Statistics (Version 29.0.0.0) rather than GNU PSPP Statistical Analysis Software.

### Eligibility criteria

Included articles were required to describe at least one human patient age 18–89 with PSPS-2 as defined previously [1], having patient(s) with axial or radicular low-back pain and previous lumbar spine surgery. Included articles were required to describe patients receiving SMT administered to any region of the spine (i.e., cervical, thoracic, lumbar) or pelvis/sacroiliac regions, such that predictors of use of lumbar SMT could be examined. Articles reporting a positive or equivocal response to SMT were included while those reporting serious adverse events were excluded.

Observational studies including case reports and series were included. Randomized controlled trials were excluded per our a priori protocol [23], as these study designs often exclude patients with previous surgery [30], treating practitioners would be less likely to use an individualized, pragmatic treatment approach tailored to the patient’s clinical and surgical characteristics, and detailed IPD such as the number of motion segments would likely be unavailable.

### Information sources

PubMed, Web of Science, Scopus, OVID/Medline, PEDro, and Index to Chiropractic Literature were searched from inception without language restrictions. Several grey literature sources were searched including National Board of Chiropractic Examiners reference text books [31], Index to Chiropractic Literature for conference material, ResearchSquare for preprints, and ProQuest for theses. Additional articles were obtained via citation tracking and contribution of articles from the personal collection of co-investigators.

### Search strategy

The search strategy included two main search themes of SMT and PSPS-2. For the PubMed search, the SMT theme included the Medical Subject Headings (MeSH) term “Musculoskeletal manipulations” as well as several other manipulation terms appearing in the title and/or abstract. The PSPS-2 theme included MeSH terms such as “Failed back surgery syndrome” and “Laminectomy” as well as several other lumbar surgical procedure terms appearing in the title and/or abstract. This search strategy was then adapted for the other databases (Additional file 1).

Articles were not excluded based on language or description of LBP severity. Google Translate was used to translate non-English abstracts and articles for the purposes of screening and data extraction. Database searches were conducted in January 2022. Articles from other sources were searched for and/or added by co-investigators in February 2022. A peer reviewer subsequently provided an additional article.

### Selection process

Two independent reviewers (RT, CD) performed initial title and abstract screening. Additional references were obtained by contacting subject matter experts, having co-investigators contribute articles to screening that were not identified by the database searches, citation tracking, and hand-searching key textbooks and prior review papers [8, 32]. Full texts were obtained and reviewed independently by two investigators (RT, CD).

Requests for de-identified IPD were made to study corresponding authors when there was insufficient data to determine if individual patients met study selection criteria [23]. This was applied to articles which reported aggregate data of patients with previous spine surgery, thus the individual patient age(s), location of surgery, and response to care could not be verified.

### Data collection process

Two investigators (RT, KM) independently extracted data from included studies into a standardized Excel workbook. Once complete, these were compared for agreement and individual discrepancies were discussed. We requested IPD from 15 corresponding authors of potentially eligible studies and were successful with obtaining IPD in two requests. Reasons for being unable to obtain IPD in 13 instances included a lack of response from the corresponding author (n = 8), the corresponding author was no longer at the institution where the study was conducted (n = 3), and the corresponding author was unable to share IPD due to a data use or ethics agreement (n = 2). As our study design required IPD, aggregate data from these 13 studies were not used.

### Data items

#### Primary outcomes

- Lumbar-SMT: Defined for the purposes of this study as SMT using a lumbar spine contact or creating movement at lumbar segments.
- Manual-thrust-SMT: Defined as SMT using an impulse or thrust, including Maitland grade V mobilizations, but excluding grade I-IV mobilizations and mechanical SMT instruments such as Activator®.
- Lumbar surgery-to-SMT interval of less than or greater than or equal to one year: Defined as the timespan between the patient’s most recent lumbar surgery and SMT application.

#### Secondary outcomes

- Provider type: Defined as the treating practitioner’s degree such as chiropractic, physical therapy, medical or osteopathic doctor, traditional East Asian medicine, or other.
- Spinal implant(s): Defined as any biomaterial introduced into the lumbar region (e.g., cage, rods, plates, spinal cord stimulator, screws, plates, disc replacement, or other).
- Post-surgical imaging: Defined as including computed tomography, magnetic resonance imaging, radiographs, and/or nuclear medicine imaging studies.
- Below-gluteal-fold symptoms: Defined as symptoms distal to this boundary.
- Number of mobile lumbar segments: Defined as any potential for segmental motion and categorized as 0, 1, 2, 3, 4, or “5 or more.” In a modification to our protocol, this variable was simplified into a reduced (0–4) or non-reduced (5) number of motion segments for the regression models only.
- SMT technique descriptions: These were extracted in free text according to what was listed in the original publication (e.g., flexion-distraction, instrument-assisted manipulation, grade III mobilization, side posture). Proprietary SMT techniques were harmonized to generic, non-proprietary terminology.

### Individual patient data integrity

Data within included articles was evaluated by three co-investigators for completeness as part of the data extraction and risk of bias assessment. IPD obtained via request were converted to Portable Document Format and appended to included article full texts.

### Study risk-of-bias assessment

Two reviewers (RT and CD) independently conducted a risk-of-bias assessment using a rubric modified from a previous study and intended for individual cases [33]. Per our a priori protocol, we aimed to apply this to individual patient cases appearing in case reports, series, or larger observational studies. Discrepancies were resolved through mutual discussion.

### Effect measures

Odds ratios were calculated for each of the primary outcomes of lumbar-SMT, lumbar-manual-thrust SMT, and lumbar surgery-to-SMT interval. The proportion and/or mean and standard deviation for each data item was calculated for the secondary outcomes.

### Synthesis methods

All cases from included articles were synthesized within the main qualitative analysis, quantitative descriptive statistical analysis, and regression models. We did not use a traditional meta-analytical approach of a two-stage pooled fixed or random effects model as our study chiefly included case reports and series, and this approach would lead to inappropriately large weighting of primary outcomes [33]. Instead, we used a one stage regression which accounted for small study size, in which the unit of measure was individual cases. This approach allowed us to examine the influence of key covariates on outcomes pertinent to our hypotheses [34].

Data items were tabulated in Microsoft Excel and those having multiple categories were displayed visually. Following the risk of bias assessment, cases missing at least 25% of the data items or having a low quality (high risk of bias) were excluded and the regression models were repeated for a sensitivity analysis.

When synthesizing surgery type, some patients had multiple distinct surgeries which were performed on the same day or over a span of several weeks, months, or years. Considering that listing each category of unique combinations of surgeries would create an excessive number of categories, the frequency of surgeries was summarized by their individual frequency or instance rather than any unique combination of several procedures.

Bivariate correlation testing was performed before logistic regression using a 2-tailed Pearson correlation matrix to identify variables with a correlation coefficient of at least 0.55. Lumbar-SMT and lumbar manual-thrust-SMT displayed a significant, moderate, positive correlation (Pearson coefficient of 0.58, *P* < 0.001). Accordingly, lumbar manual-thrust-SMT was excluded from two regression models as an independent variable, while lumbar-SMT was excluded as an independent variable in the regression wherein lumbar manual-thrust-SMT was the dependent variable. Three multiple binary logistic regression models were conducted:

1. The dependent variable was lumbar-SMT (used vs. not used), with covariates including: patient age, below-gluteal-fold symptoms, motion segments, spinal implant, post-surgical imaging, provider type, and surgery-to-SMT interval.
2. The dependent variable was lumbar manual-thrust-SMT (used vs. not used), with covariates including: patient age, below-gluteal-fold symptoms, motion segments, spinal implant, post-surgical imaging, provider type, and surgery-to-SMT interval.
3. The dependent variable was the surgery-to-SMT interval (< 1 or ≥ 1 year), with covariates including: patient age, below-gluteal-fold symptoms, motion segments, spinal implant, provider type, post-surgical imaging, and lumbar-SMT.

Missing data were treated as a separate category in regression analysis. Cases in which the dependent variable was missing could not be included the binary logistic regression models. This only slightly reduced the effective sample size for each regression model (i.e., n = 97 cases in model 1, n = 96 in model 2, n = 95 in model 3) which remained above the level of minimum sample size threshold.

### Exploration of variation in effects

This step was not applicable as our study examined treatment patterns rather than effects of care.

### Risk of bias across studies

Our strategy to assign a separate category to missing or unclear values in regression analysis is a valid [35], yet can bias results. We examined the possible bias introduced by this method via a sensitivity analysis in which cases missing at least 25% of data items or having a high risk of bias (low quality) were excluded and regressions were then repeated.

## Results

### Study selection

**Fig. 1:**
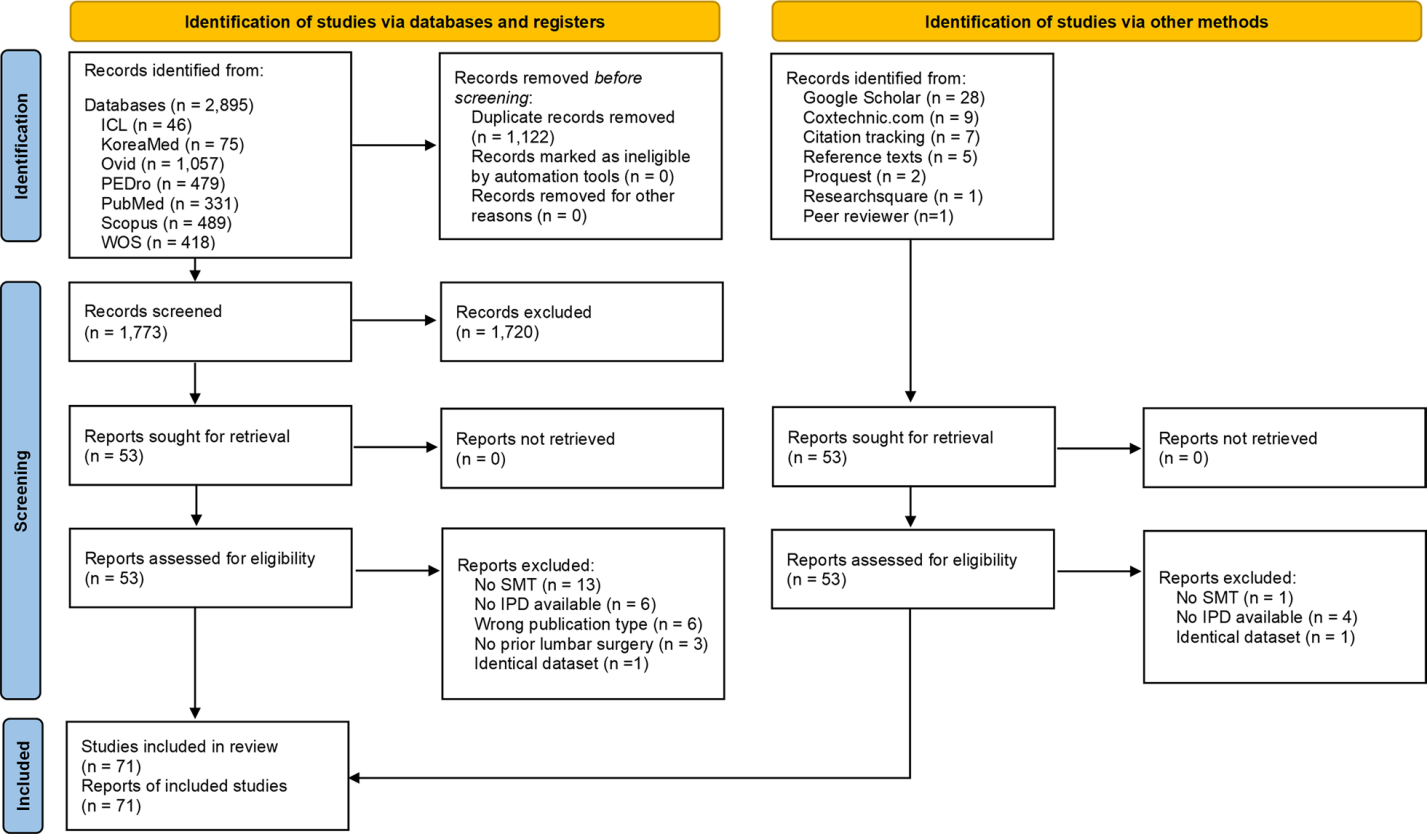
PRISMA 2020 flow diagram including searches of databases, registers, and other sources. Abbreviations: Index to Chiropractic Literature (ICL), individual patient data (IPD), Physiotherapy Evidence Database (PEDro), spinal manipulative therapy (SMT), Web Of Science (WOS)

After removal of duplicates, the search identified 1,825 articles from databases and other sources. After conducting article screening, 71 articles were included in this review. describing 103 individual patients (Fig. 1) [36–106].

### Study characteristics

Of the 71 included studies, 59 described a single patient while 11 described more than one patient. The mean number of patients per study was 1.5 [SD] ± 1.5. Nine studies appeared to meet the selection criteria but were excluded as IPD were not available [72, 107–115]. An abbreviated form of the included studies and cases, which omits the study title, and free text descriptions of surgery and SMT, is shown in Table 1 while the full dataset is included in Additional file 2.

**Table 1:** Included articles with corresponding cases

Author	Year	Patient age	Patient Sex	Symptoms distal to gluteal fold	Treating provider profession	Number of motion segments 0–5	Presence of spinal implant	Presence of post-surgical imaging	Lumbar SMT reported	Lumbar manual thrust SMT reported	Surgery to SMT interval (± 1 year)
Adams [36]	2004	41	F	Y	Chiro	5	N	Y	U	U	< 1
Alexander [37]	1993	45	M	Y	Chiro	5	N	Y	Y	Y	≥ 1
Aspegren [38]	1997	35	M	Y	Chiro	5	N	Y	Y	N	< 1
Benningfield [39]	1997	39	M	Y	Chiro	5	N	N	U	U	≥ 1
Blum [40]	2002	39	F	U	Chiro	2	Y	Y	Y	N	≥ 1
Chu [41]	2017	44	F	Y	Chiro	5	N	Y	Y	Y	≥ 1
Chu [41]	2017	44	F	Y	Chiro	5	N	Y	Y	Y	≥ 1
Clark [42]	2019	73	F	Y	Chiro	U	U	U	U	U	< 1
Cole [43]	2020	70	M	Y	Chiro	5	N	Y	Y	N	≥ 1
Colloca [44]	2003	49	F	Y	Chiro	0	Y	Y	N	N	≥ 1
Cook [45]	2021	28	F	Y	Chiro	5	N	Y	Y	N	< 1
Coulis [46]	2013	31	M	Y	Chiro	5	N	Y	Y	N	≥ 1
Coulis [46]	2013	60	M	Y	Chiro	5	N	Y	Y	Y	≥ 1
Cox [47]	2009	48	F	Y	Chiro	3	Y	Y	Y	N	≥ 1
Cox [48]	2011	43	M	Y	Chiro	5	N	Y	Y	N	U
Cox [49]	2011	27	M	Y	Chiro	5	N	Y	Y	N	U
Cox [51]	2011	43	M	Y	Chiro	3	Y	Y	Y	N	U
Cox [50]	2011	24	F	Y	Chiro	U	Y	Y	Y	N	U
Davis [52]	1993	48	M	Y	Chiro	5	N	Y	Y	Y	≥ 1
Davis [52]	1993	50	M	Y	Chiro	U	Y	Y	Y	Y	≥ 1
Dean [53]	2017	47	F	Y	Chiro	5	N	Y	Y	N	< 1
Demetrious [54]	2007	48	M	Y	Chiro	2	Y	Y	Y	N	U
Diakow [55]	1983	48	M	N	Chiro	1	Y	Y	Y	Y	< 1
Edwards [56]	2017	28	F	N	Chiro	2	Y	Y	Y	Y	≥ 1
Eisenberg, Vulfsons [57]	2011	37	M	Y	MD/DO	5	N	N	Y	N	< 1
Estadt [58]	2004	54	M	Y	Chiro	5	N	N	Y	Y	< 1
Francio [59]	2017	42	M	Y	U	5	N	Y	Y	Y	≥ 1
Frank [60]	2011	54	M	Y	Chiro	5	N	Y	Y	N	≥ 1
Gangemi [61]	2003	35	M	Y	Chiro	5	N	N	Y	N	< 1
Gluck [62]	1996	41	M	Y	Chiro	5	N	Y	Y	N	≥ 1
Greenwood [63]	2011	55	M	Y	Chiro	5	N	Y	Y	N	≥ 1
Greenwood [64]	2012	34	M	N	Chiro	2	Y	Y	Y	N	≥ 1
Hazen [65]	2015	59	M	Y	Chiro	5	N	Y	Y	N	≥ 1
Hoiriis [66]	1989	40	M	Y	Chiro	5	N	Y	N	N	≥ 1
Hong [67]	2009	46	M	Y	TEAM	5	N	Y	Y	N	U
Johnson [68]	2020	69	F	U	Chiro	U	Y	Y	Y	N	≥ 1
Kang [69]	2010	37	M	Y	TEAM	4	Y	Y	Y	N	≥ 1
Kinney [70]	2016	27	M	Y	Chiro	U	Y	U	U	U	U
Klaus [71]	2022	32	M	Y	Chiro	5	N	U	Y	N	≥ 1
Kruse [72]	2011	55	M	Y	Chiro	4	Y	Y	Y	N	≥ 1
Lamb [73]	1997	57	F	Y	Chiro	5	N	N	U	U	≥ 1
Lapham-Yaun [74]	2019	62	F	Y	Chiro	4	Y	Y	N	N	≥ 1
Layton [75]	2009	58	M	Y	Chiro	5	N	Y	Y	Y	≥ 1
Lee [76]	2010	28	M	Y	TEAM	5	N	Y	Y	N	≥ 1
Lee [76]	2010	27	M	Y	TEAM	5	N	Y	Y	N	≥ 1
Lee [76]	2010	47	F	Y	TEAM	5	Y	Y	Y	N	U
Lee [77]	2015	57	F	Y	TEAM	5	N	Y	N	N	< 1
Lee [77]	2015	43	F	Y	TEAM	5	N	Y	N	N	< 1
Lewis [78]	2017	50	F	Y	MD/DO	2	Y	Y	Y	N	≥ 1
Lim [79]	2011	70	F	Y	TEAM	4	Y	Y	Y	N	< 1
Lisi [80]	2004	35	F	N	Chiro	5	N	N	Y	Y	< 1
Loschiavo [81]	2012	66	F	Y	Chiro	4	Y	Y	U	U	U
Maddalozzo [82]	2018	45	F	Y	Chiro	3	Y	Y	Y	U	≥ 1
McGregor [83]	1983	26	M	Y	Chiro	5	N	Y	Y	Y	< 1
McGregor [83]	1983	64	F	Y	Chiro	5	N	Y	Y	Y	≥ 1
McGregor [83]	1983	61	F	Y	Chiro	4	Y	Y	Y	Y	≥ 1
Moran [84]	2008	65	M	Y	Chiro	4	N	Y	Y	N	< 1
Morningstar [85]	2012	51	M	Y	Chiro	5	N	Y	Y	Y	≥ 1
Morningstar [85]	2012	47	F	Y	Chiro	5	N	Y	Y	Y	≥ 1
Morningstar [85]	2012	64	M	U	Chiro	5	N	Y	Y	Y	≥ 1
Oakley [86]	2007	35	M	Y	Chiro	5	N	Y	Y	Y	< 1
O'Connor [87]	2021	65	F	Y	PT	0	Y	N	N	N	≥ 1
Olding [88]	2016	51	F	Y	Chiro	5	N	N	Y	N	< 1
O'Shaughnessy [89]	2010	55	M	U	Chiro	5	Y	Y	Y	Y	< 1
O'Shaughnessy [89]	2010	52	M	U	Chiro	5	Y	Y	Y	Y	< 1
O'Shaughnessy [89]	2010	44	M	U	Chiro	5	Y	Y	Y	Y	< 1
O'Shaughnessy [89]	2010	35	M	U	Chiro	5	Y	Y	Y	Y	< 1
O'Shaughnessy [89]	2010	34	M	U	Chiro	5	Y	Y	Y	Y	< 1
O'Shaughnessy [89]	2010	54	F	U	Chiro	5	Y	Y	Y	Y	< 1
O'Shaughnessy [89]	2010	46	F	U	Chiro	5	Y	Y	Y	Y	< 1
O'Shaughnessy [89]	2010	55	M	U	Chiro	5	Y	Y	Y	Y	≥ 1
Paris [90]	2017	59	M	Y	PT	4	Y	Y	Y	N	≥ 1
Perrucci [91]	2017	73	M	Y	Chiro	4	Y	Y	Y	N	≥ 1
Perrucci [91]	2017	58	M	Y	Chiro	3	Y	Y	Y	Y	≥ 1
Perrucci [91]	2017	81	M	N	Chiro	4	Y	Y	Y	Y	≥ 1
Perrucci [91]	2017	57	M	Y	Chiro	5	Y	Y	Y	Y	≥ 1
Peterson [92]	2016	72	F	Y	PT	5	Y	N	Y	N	≥ 1
Puentedura [93]	2010	50	M	N	PT	5	Y	N	Y	N	< 1
Roloff [94]	2020	37	F	Y	Chiro	5	N	Y	Y	Y	< 1
Romano-Young [95]	2017	67	F	N	Chiro	5	N	U	U	U	< 1
Schultz [96]	2017	45	F	Y	PT	5	N	N	Y	N	≥ 1
Schwab [97]	2008	47	M	Y	Chiro	5	N	Y	Y	Y	≥ 1
Seo [98]	2015	77	M	Y	TEAM	5	N	Y	Y	N	< 1
Seo [98]	2015	58	M	Y	TEAM	5	N	Y	Y	N	< 1
Seo [98]	2015	73	M	Y	TEAM	5	N	Y	Y	N	≥ 1
Seo [98]	2015	72	M	Y	TEAM	5	N	Y	Y	N	≥ 1
Seo [98]	2015	81	F	Y	TEAM	5	N	Y	Y	N	≥ 1
Seo [98]	2015	80	F	Y	TEAM	5	N	Y	Y	N	≥ 1
Seo [98]	2015	65	F	Y	TEAM	3	Y	Y	Y	N	≥ 1
Seo [98]	2015	78	F	Y	TEAM	4	Y	Y	Y	N	≥ 1
Seo [98]	2015	77	F	Y	TEAM	4	Y	Y	Y	N	≥ 1
Seo [98]	2015	75	F	Y	TEAM	5	Y	Y	Y	N	≥ 1
Seo [99]	2016	77	F	Y	TEAM	4	Y	Y	Y	N	≥ 1
Seo [99]	2016	77	F	Y	TEAM	4	Y	Y	Y	N	≥ 1
Seo [99]	2016	75	F	Y	TEAM	4	Y	Y	Y	N	≥ 1
Seo [99]	2016	55	F	Y	TEAM	4	Y	Y	Y	N	≥ 1
Shaw [100]	1996	33	F	Y	Chiro	5	N	Y	Y	Y	≥ 1
Siciliano [101]	2019	71	M	Y	Chiro	5	Y	Y	Y	N	≥ 1
Taylor [102]	2007	67	F	Y	Chiro	5	N	N	Y	N	≥ 1
Toomey [103]	2020	44	F	Y	PT	4	Y	Y	Y	Y	≥ 1
Torres [104]	2017	51	M	N	PT	3	Y	N	N	N	< 1
Vaillancourt [105]	1993	33	M	Y	Chiro	3	Y	Y	N	N	≥ 1
Welk [106]	2012	24	M	N	Chiro	5	N	Y	Y	N	≥ 1

*Chiro* chiropractic, *DO* doctor of osteopathy, *F* female, *M* male, *MD* medical doctor, *N* no, *PT* physical therapist, *SMT* spinal manipulative therapy, *TEAM* traditional East Asian medicine practitioner, *Y* yes, *U* unclear

During the database title and abstract screening there were 52 discrepancies (97% agreement between reviewers) which were all resolved via discussion. Seven articles were kept as a “maybe” until the IPD request process was complete, then either included or excluded depending on the information provided. During the database full text screening from other sources there were two discrepancies (96% agreement) which were resolved via discussion. During title and abstract screening from other sources there were six discrepancies which were resolved via discussion (89% agreement), while four articles were kept as “maybe” until the IPD process was complete.

### Individual patient data integrity

IPD requests were successful in two articles which allowed inclusion of both in the current review while the remainder were excluded. There were 90 data items in disagreement during the initial data extraction (93% agreement), 87 of which were resolved via discussion. For one article this required re-translation of a section of text. Another disagreement was resolved after obtaining an article describing the SMT technique used by the authors. Only three disagreements were adjudicated by a third investigator, all of which related to the number of motion segments in each patient [84, 92, 101].

### Risk of bias in studies

There were seven discrepancies in the initial risk of bias assessments (98% agreement) which were all resolved via discussion. This process resulted in three cases being deemed low quality (3%), eight being moderate quality (8%), and the 92 remaining (89%) being high quality. Scores per each case are included in Additional file 2.

### Results of individual studies

The results in this review are presented in terms of individual patients combined in a one-stage approach, rather than summarized at the study level. Accordingly, this section does not apply to the current review.

### Results of syntheses

#### Clinical features

Of the 103 patients the mean age [SD] was 51.5 ± 15.3, with 55% of the population being male and the remainder female. Most patients (81%) had symptoms distal to the gluteal fold, while nine percent had symptoms proximal to the gluteal fold, and this data item was unclear in the remainder.

#### Surgical features

**Fig. 2:**
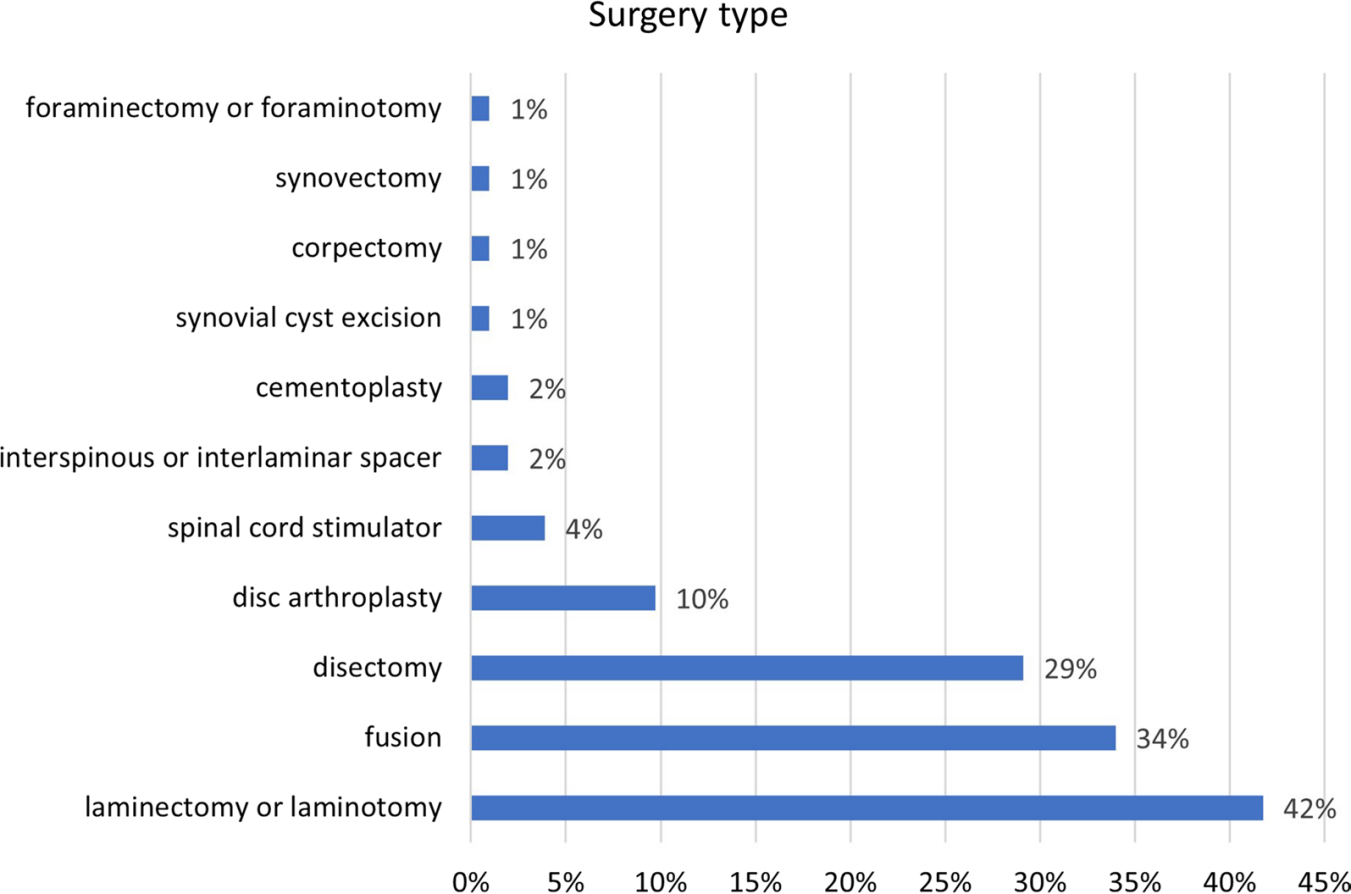
Types of surgery by instance

There were 130 distinct surgical procedures per 103 patients. Laminectomy or laminotomy was the most common, occurring in 43 instances, followed by fusion (35), discectomy (30), disc arthroplasty (10), spinal cord stimulator (4), and other less common surgeries (Fig. 2). Twenty-three patients had only a laminectomy or laminotomy and no other surgical procedure, while 10 patients had a laminectomy as well as another surgical procedure, such as a discectomy or fusion.

**Fig. 3:**
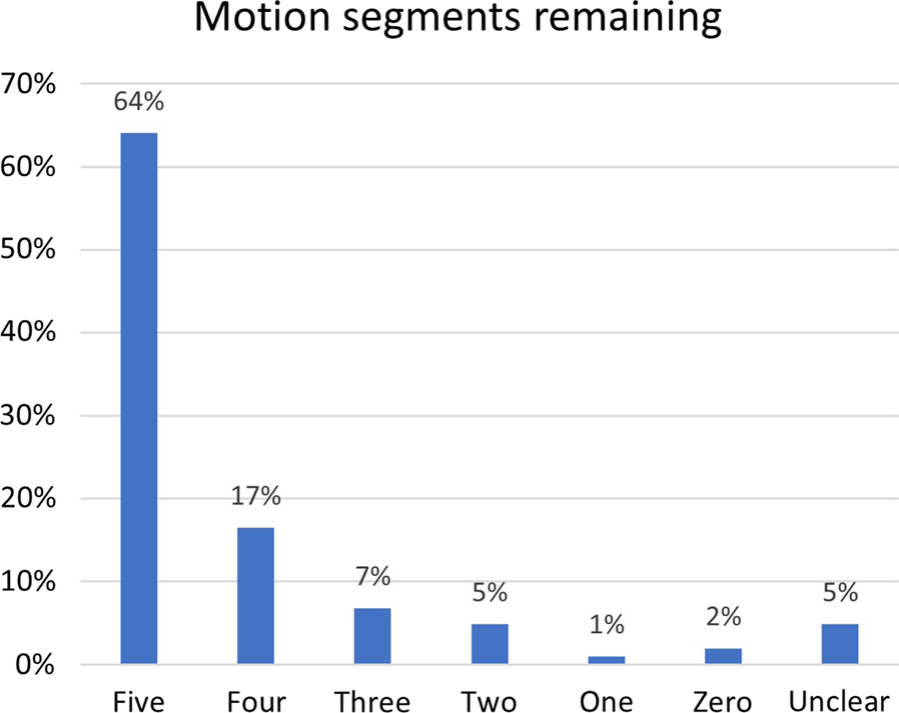
Number of lumbar motion segments remaining following lumbar spine surgery

Over half of patients (64%) had a normal number of motion segments following lumbar spine surgery (i.e., five or more) while 31% had a reduced number of motion segments (i.e., < 5). This data item was unclear in five percent of patients (Fig. 3). About half of patients had a spinal implant (48%) or had no implant (51%) while this data item was unclear in one patient (1%). Post-surgical imaging was available to the SMT practitioner in 83% of cases, was not available to practitioners in 13% of cases, and this data item was unclear in four percent.

#### Spinal manipulative therapy

**Fig. 4:**
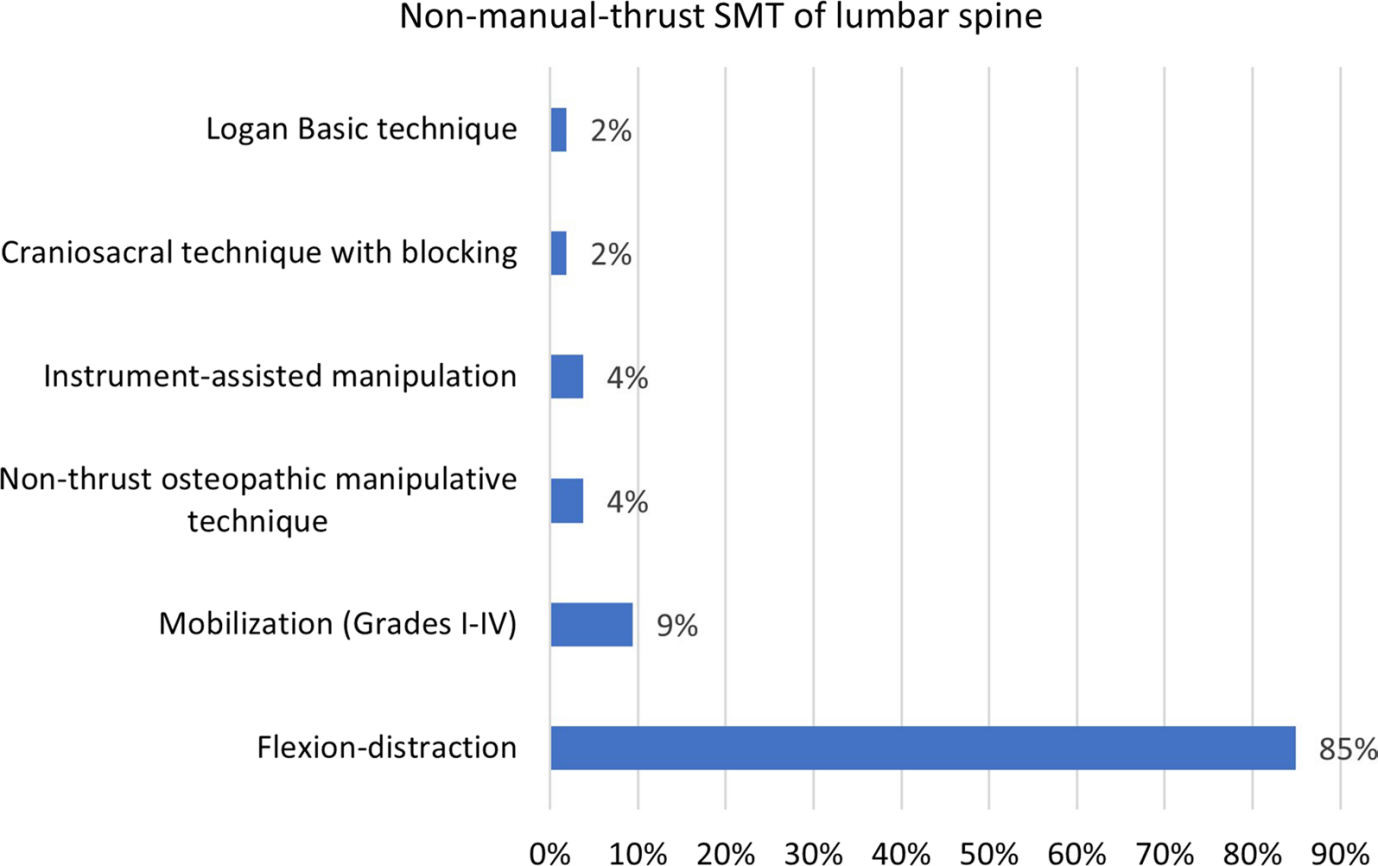
Subgroup analysis of 53 cases in which a non-manual-thrust-SMT technique was applied to the lumbar spine

The most common type of practitioner administering SMT was a chiropractor (69%), followed by TEAM practitioner (21%), physical therapist (7%), and MD or DO (2%). This data item was unclear in a single case (1%). Lumbar SMT was administered in 85% of cases (i.e., 88 patients) and was not administered in eight percent, while this data item was unclear in seven percent of cases. Of the 88 patients receiving lumbar SMT, manual thrust SMT was not used in 69% of cases (i.e., 69 patients), while manual-thrust-SMT was used in 39%. The most frequently applied lumbar non-manual-thrust SMT technique was flexion-distraction (85% of these cases), followed by Grade I-IV mobilization (9%) (Fig. 4). The percentage of lumbar SMT that involved manual thrust varied per provider type, being highest among chiropractors (53% of instances of lumbar SMT involved thrust), followed by physical therapists (20%), while MD/DO and TEAM providers did not use lumbar manual-thrust-SMT.

Spinal manipulative therapy was administered within one year after surgery in 29% of cases and was administered in greater than one year following surgery in 62% of cases. This data item was unclear in nine percent of cases.

There were eight cases in which no lumbar manipulation was provided. In four of these cases the practitioner used an SMT technique which was, according to the definition in our original protocol [23], isolated to the sacroiliac joint or pelvis [44, 74, 77]. In the other four, SMT was directed to the cervical and/or thoracic regions [66, 87, 104, 105]. There were six cases in which providers used lumbar thrust SMT in patients with a reduced number of lumbar motion segments who also had a spinal implant [55, 56, 83, 91, 103]. Of the patients with only a laminectomy or laminotomy and no other surgical procedure (n = 23), 96% received lumbar SMT, while 35% received lumbar-manual-thrust-SMT.

#### Regression models

Each of the three binary logistic regression models did not yield any statistically significant odds ratios for our primary outcomes (i.e., P > 0.05 for each; Table 2). However, a non-reduced number of motion segments approached significance for predicting use of lumbar manual-thrust-SMT (OR 9.07 [0.97–84.64], *P* = 0.053). The hypothesis for our secondary outcome was supported as chiropractors were more likely to use lumbar manual-thrust-SMT (OR 32.26 [3.17–327.98], *P* = 0.003).

**Table 2:** Key results of the binary logistic regression models

Independent variable	Dependent variable					
	Lumbar SMT		Lumbar thrust SMT		Time to SMT < 1 year	
	Odds ratio (95% CI)	*P*-value	Odds ratio (95% CI)	*P*-value	Odds ratio (95% CI)	*P*-value
Patient age	1.05 (0.98–1.12)	0.195	1.00 (0.96–1.05)	0.954	1.03 (0.99–1.07)	0.176
Patient sex female (ref. male)	0.51 (0.09–2.79)	0.435	1.47 (0.45–4.84)	0.529	0.80 (0.26–2.41)	0.797
No radiation below gluteal fold (ref. radiation below gluteal fold)	2.97 (0.17–53.52)	0.461	3.28 (0.45–23.63)	0.239	0.14 (0.02–1.03)	0.138
Chiropractor (ref. non chiropractor)	1.42 (0.24–8.31)	0.701	32.26 (3.17–327.98)	0.003*	1.94 (0.50–7.51)	0.339
No implant (ref. implant)	1.04 (0.01–75.59)	0.985	0.27 (0.03–2.20)	0.789	0.43 (0.05–3.84)	0.449
Motion segments non-reduced (ref. reduced)	7.60 (0.14–406.79)	0.318	9.07 (0.97–84.64)	0.053	0.33 (0.35–3.19)	0.340
Post-surgical imaging (ref. no)	2.99 (0.34–26.06)	0.323	4.09 (0.47–35.83)	0.203	2.42 (0.57–10.20)	0.228
Lumbar SMT (ref. no)	NA	NA	NA	NA	2.26 (0.35–14.76)	0.395
Time to SMT > 1 year (ref. ≤ 1 year)	2.73 (0.33–23.01)	0.355	0.64 (0.15–2.82)	0.557	NA	NA

^*^Bold value indicates *P* < 0.05

### Risk of bias across studies

According to the sensitivity analysis protocol [23], cases missing at least 25% of the data items (three cases), or having a high risk of bias (low quality), were excluded, resulting in the omission of four cases from the dataset. Results for all outcomes were similar (Additional file 3). For the regression models having lumbar-SMT and lumbar manual-thrust-SMT as the dependent variable, results were unchanged given identical cases had been effectively omitted as separate categories when they were unclear in the initial regression models. Results for timing as a dependent variable were slightly different yet remained non-significant.

### Risk of bias related to unavailable IPD

Omission of studies with unavailable IPD could have influenced the results of this study as several potentially eligible cases could not be obtained (Table 3). Although there were two large series of patients receiving a potentially multimodal SMT approach from multiple providers [107, 109], there were also two large series of patients exclusively treated using non-manual-thrust SMT (flexion-distraction) [72, 108]. While these studies included a large quantity of data, it is unclear if addition of cases from these studies would act to support or contradict our study hypotheses. Regardless, additional cases could have enabled us to reach statistical significance in our regression models given our sample size was just above the minimum threshold.

**Table 3:** Studies potentially meeting selection criteria yet not included due to unavailable individual patient data

Author	Year	Patients (n)	Patient characteristics	SMT practitioner	SMT description
Pfefer [107]	2012	31	"failed back surgery," otherwise unclear	Chiropractor	Multimodal
Gudavalli [108]	2016	69	Age (mean): 61 Sex: 58% female Surgeries: Discectomy 58%, fusion 38%	Chiropractor	Non-manual-thrust: Flexion-distraction
Lee [109]	2017	120	Age (mean): 41.9 ± 11.7 Sex: 60% male Most common surgery: Laminectomy 83%	TEAM	Multimodal
Kruse [72]	2011	31	Age (mean): 50.6 ± 9.8 Sex: 50% female	Chiropractor	Non-manual-thrust: Flexion-distraction
Park [110]	2021	106	Age (mean): 54.9 ± 11.5 Sex: 60% female Most common surgeries: Discectomy (41%), laminectomy (31%)	TEAM	Unclear
Taber [111]	2014	Unclear	"Lumbar post-laminectomy syndrome," otherwise unclear	Chiropractor	Manual-thrust
Stern [112]	1995	8	Previous history of lumbar spine surgery, otherwise unclear	Chiropractor	Unclear
Wirth [113]	2019	12	Previous back surgery, otherwise unclear	Chiropractor	Unclear
Fruhwirth [114]	1992	106	"postoperative vertebral pain symptoms," otherwise unclear	Chiropractor	Unclear

*SMT* Spinal manipulative therapy, *TEAM* Traditional East Asian medicine practitioner

## Discussion

### Summary of evidence

In the first study to examine IPD in patients receiving SMT for PSPS-2, we found that most cases reported use of non-manual-thrust lumbar SMT, and chiropractors were more likely to use manual-thrust lumbar SMT relative to other provider types. These findings suggest that practitioners generally use a cautious approach to SMT in those with previous lumbar spine surgery, however, providers using lumbar thrust-SMT for PSPS-2 are more likely to be chiropractors. These findings refute our primary hypothesis that the SMT approach in PSPS-2 is predicted by markers of clinical or surgical complexity, and instead support our secondary hypothesis that use of thrust-SMT is predicted by provider type.

Non-thrust SMT is typically described as a gentler technique, more appropriate when there are precautions such as osteoporosis or hypermobility [12, 116]. As an individual variable, the force used in SMT does not distinguish thrust from non-thrust techniques [13]. However, in practice, the mean forces used by providers in the lumbopelvic region do vary between these approaches. A systematic review found that the mean peak force applied to the lumbar spine in Newtons (N) ranged from 210 to 495 N in thrust-SMT, while another study reported a mean of 102 N for flexion distraction technique [117], the most common non-thrust SMT identified in this review. It is possible that clinicians, when wary of potential complications, default to a gentler technique given the outcomes between thrust and non-thrust SMT may be similar [118].

This study suggests that while providers generally use non-thrust rather than thrust-SMT in the lumbar region when treating those with PSPS-2, chiropractors are significantly more likely to use thrust-SMT than other provider types. Evidence suggests that chiropractors generally use thrust SMT more frequently than other practitioners [11, 119]. A recent scoping review found that 80% of chiropractic encounters included manual or assisted thrust SMT whereas only 17% included non-thrust SMT or traction [120]. In contrast, one study reported that only 3–14% of physical therapists used thrust SMT to manage non-specific low back pain [121]. While we are unaware of the general frequency of use of thrust versus non-thrust SMT among MD, DO and TEAM providers, none of the cases with these provider types in the current study reported use of lumbar manual-thrust-SMT. Our study design does not permit us to specify which form of lumbar SMT (i.e., thrust- or non-thrust) is more appropriate, safer, or effective in patients with PSPS-2.

Our regression model found that a non-reduced number of motion segments (e.g., no surgical fusion) approached significance for predicting an increased odds of using lumbar-manual-thrust SMT (OR 9.07 [0.97–84.64], *P* = 0.053). While not statistically significant, this finding does not rule out a clinically important effect of the number of motion segments on providers’ choice of SMT use. Given that a small sample size likely contributed to lack of statistical significance (i.e., *P* > 0.05), it remains possible that providers are more likely to use thrust-SMT on the lumbar spine of a patient with PSPS-2 having a normal number of motion segments (e.g., discectomy, laminectomy) in contrast to a patient with a reduced number of motion segments (e.g., surgical fusion).

A small percentage of providers addressed PSPS-2 by focusing SMT only on non-lumbar regions including the cervical and thoracic spine or sacroiliac joint. Given that SMT may produce beneficial effects regardless of the exact site of application [16], it is possible SMT providers in these included cases were attempting to take advantage of a nonspecific hypoalgesic effect of SMT. Considering we only included cases with a positive or equivocal outcome, all SMT approaches identified in this review are potential options for such patients. This review highlights that a variety of SMT approaches are used in patients with several distinct types of prior surgery.

This review reinforces that further research is needed regarding SMT for patients with PSPS-2. Comparative effectiveness trials could be used to compare SMT approaches or compare SMT to exercise and/or medications. The overall effectiveness of SMT for PSPS-2 also needs further research with respect to several outcomes, including safety of SMT, changes in patient-reported disability and pain, likelihood of medication use, epidural steroid injections, revision surgery, or other procedures. Further, qualitative studies or surveys examining practitioners’ choice towards certain SMT techniques in patients with PSPS-2 are needed, examining not only the variables in this study but also patient and practitioner preferences.

### Strengths and limitations

The current study is the first to examine individual patient-level features of patients with PSPS-2 and how these relate to the application of SMT. Our study utilized an extensive search strategy including grey literature, as well as submitting IPD requests to obtain further data. Several data items were obtained, and extraction and risk of bias were conducted in duplicate to reduce errors.

This review has several limitations. First, limited data granularity in included cases required omitting several potentially relevant data items which would be unlikely to appear in a publication. Practitioners’ choice of SMT technique may depend on patients’ preference to a certain technique or previous adverse experience with a certain technique, which were not considered in this review. Further, bone density, patients’ response to pre-manipulative loading testing (i.e., to determine tolerance to SMT), and their ability to be positioned comfortably on the treatment surface may factor into practitioners’ decision-making for SMT application. Such variables may be better explored by qualitative interviews or international surveys regarding SMT selection for PSPS-2 based on hypothetical patient scenarios.

This review did not examine practitioner-related variables such as specific instructions provided by a referring surgeon, fear of litigation related to patient injury, or available equipment in the SMT providers’ office which may have guided practitioners towards a particular SMT strategy. Further, some practitioners may have used acupuncture, nutritional supplements, exercises, or other modalities in addition to or in place of lumbar manual thrust SMT. Extraction of these variables was not feasible as these were heterogeneous and/or inconsistently reported.

While our protocol aimed to avoid including cases with protocol-driven care, we included multiple case series that appeared to apply an identical SMT approach (i.e., regarding lumbar and lumbar-manual-thrust variables) to all patients regardless of clinical features. This tendency could also be a form of publication bias in the sense that authors could group patients receiving a certain treatment together for a more cohesive or presentable case series. Considering a relatively arbitrary threshold of articles describing at least three patients, this accounted for 31 cases in our review in which clinical variables may not have been the driving force for treatment selection [76, 83, 85, 89, 98, 99]. An abundance of case series describing a similar SMT approach for all patients is suggestive of a practitioner-driven tendency towards using a certain type of SMT, rather than the individualized approach we hypothesized.

Our review did not analyze patient outcomes, as these were inconsistently reported and not feasible to synthesize. Further, this was considered to not be appropriate given included articles were case reports and series, subject to potential publication bias for cases with a more positive outcome. However, if larger studies were available, it could allow for a more in-depth analysis of the association between SMT technique and patient outcomes in PSPS-2.

The included sample of published cases may not necessarily reflect what is generally done by SMT practitioners worldwide. Very few cases of SMT applied by MDs/DOs were identified. Given the larger series with unavailable IPD, it is possible that a broader variety of SMT approaches were not captured in our study. Although we did not extract data regarding author affiliations, it remains possible that published cases were more often linked to educational institutions.

Although IPD systematic reviews with meta-analyses are considered a gold standard of evidence synthesis [24], the certainty of evidence of this review remains low given that it is based on case reports and series [33].

### Conclusions

Practitioners tend to use potentially gentler non-thrust SMT techniques in patients with PSPS-2, most frequently flexion-distraction, and often opt to avoid manual thrust SMT in the lumbar spine. Although lumbar-manual-thrust SMT is not often used in PSPS-2, chiropractors are more likely to use this form of treatment relative to other provider types. It is possible that unmeasured variables, such as patient or provider preferences, are more predictive of the SMT approach in those with PSPS-2 than those examined in the current review, or that limited sample size influenced our findings.

## Supplementary Information

**Additional file 1**. Search strategies.

**Additional file 2**. Raw data.

**Additional file 3**. Sensitivity analysis.

## Data Availability

All data generated or analysed during this study are included in this published article and its Additional files.

## Abbreviations

FBSS: Failed back surgery syndrome
HVLA: High-velocity, low-amplitude
ICL: Index to Chiropractic Literature
IPD: Individual participant data
PRISMA-IPD: Preferred Reporting Items for a Systematic Review and Meta-analysis of Individual Participant Data
PRISMA-P: Preferred reporting items for systematic review and meta-analysis protocols
PROSPERO: International Prospective Register of Systematic Reviews
PSPS-2: Persistent spinal pain syndrome type 2
SMT: Spinal manipulative therapy
TEAM: Traditional East Asian Medicine
WOS: Web of Science
