# Supplementary material for: Clinician approaches to spinal manipulation for persistent spinal pain after lumbar surgery: systematic review and meta-analysis of individual patient data
Source: Chiropr Man Therap. 2023 Mar 9;31:10. doi: 10.1186/s12998-023-00481-5 (PMC9999664; doi:10.1186/s12998-023-00481-5)
Supplement: Supplementary file 1 — Additional file 1. Search strategies. [file 12998_2023_481_MOESM1_ESM.docx]

# Supplemental File 1 - Search Strategies

**PUBMED**

**1/6/2022**

Search number #, Query, **Results**

75 (((((((((((((((((((((((((((((((tuina[tiab]) OR ("tui na"[tiab])) OR (chuna[tiab])) OR (flexion

distraction[tiab])) OR (cox technique[tiab])) OR (hvla[tiab])) OR (high velocity low amplitude[tiab])) OR (instrument assisted adjust*[tiab])) OR (instrument assisted manipulation*[tiab])) OR (joint manipulation*[tiab])) OR (joint mobilization*[tiab])) OR (joint mobilisation*[tiab])) OR (manipulation under anesthesia[tiab])) OR (manipulation under anaesthesia[tiab])) OR (fibrosis release[tiab])) OR ("musculoskeletal manipulations"[mesh])) OR (musculoskeletal manipulation*[tiab])) OR (manipulative rehabilitation*[tiab])) OR (manipulative therap*[tiab])) OR (manual therap*[tiab])) OR (mobilization therap*[tiab])) OR (mobilisation therap*[tiab])) OR (orthopedic manipulation*[tiab])) OR (orthopaedic manipulation*[tiab])) OR (osteopathic manipulation*[tiab])) OR (chiropractic manipulation*[tiab])) OR (soft tissue mobilization*[tiab])) OR (soft tissue mobilisation*[tiab])) OR (spinal manipulation*[tiab])) OR (spinal mobilization*[tiab])) OR (spinal mobilisation*[tiab])) AND ((((((((((((((((((((((((((((((((((((((((("total disc replacement"[mesh]) OR (disc replacement*[tiab])) OR (disk replacement*[tiab])) OR (disc arthroplast*[tiab])) OR (disk arthroplast*[tiab])) OR (cementoplasty[mesh])) OR (cementoplast*[tiab])) OR (vertebroplast*[tiab])) OR (kyphoplast*[tiab])) OR (diskectomy[mesh])) OR (discectom*[tiab])) OR (diskectom*[tiab])) OR (extreme lateral interbody fusion*[tiab])) OR (xlif[tiab])) OR (foraminotomy[mesh])) OR (foraminotom*[tiab])) OR (interlaminar lumbar instrumented fusion*[tiab])) OR (ilif[tiab])) OR (interlaminar implant*[tiab])) OR (intrathecal drug delivery[tiab])) OR (itdd[tiab])) OR (laminectomy[mesh])) OR (laminectom*[tiab])) OR (laminotom*[tiab])) OR (laser surger*[tiab])) OR (microdiskectom*[tiab])) OR (microdiscectom*[tiab])) OR (persistent spinal pain syndrome[tiab])) OR (postoperative[tiab])) OR (post-operative[tiab])) OR (postsurgical[tiab])) OR (post-surgical[tiab])) OR ("spinal cord stimulation"[mesh])) OR (spinal cord stimulat*[tiab])) OR (adjacent segment disease[tiab])) OR ("failed back surgery syndrome"[mesh])) OR (failed back surgery syndrome*[tiab])) OR (fbss[tiab])) OR (junction failure*[tiab])) OR (recurrent disc*[tiab])) OR (recurrent disk*[tiab])) **1,085**

74 (((((((((((((((((((((((((((((((((((((((("total disc replacement"[mesh]) OR (disc

replacement*[tiab])) OR (disk replacement*[tiab])) OR (disc arthroplast*[tiab])) OR (disk arthroplast*[tiab])) OR (cementoplasty[mesh])) OR (cementoplast*[tiab])) OR (vertebroplast*[tiab])) OR (kyphoplast*[tiab])) OR (diskectomy[mesh])) OR (discectom*[tiab])) OR (diskectom*[tiab])) OR (extreme lateral interbody fusion*[tiab])) OR (xlif[tiab])) OR (foraminotomy[mesh])) OR (foraminotom*[tiab])) OR (interlaminar lumbar instrumented fusion*[tiab])) OR (ilif[tiab])) OR (interlaminar implant*[tiab])) OR (intrathecal drug delivery[tiab])) OR (itdd[tiab])) OR (laminectomy[mesh])) OR (laminectom*[tiab])) OR (laminotom*[tiab])) OR (laser surger*[tiab])) OR (microdiskectom*[tiab])) OR (microdiscectom*[tiab])) OR (persistent spinal pain syndrome[tiab])) OR (postoperative[tiab])) OR (post-operative[tiab])) OR (postsurgical[tiab])) OR (post-surgical[tiab])) OR ("spinal cord stimulation"[mesh])) OR (spinal cord stimulat*[tiab])) OR (adjacent segment disease[tiab])) OR ("failed back surgery syndrome"[mesh])) OR (failed back surgery syndrome*[tiab])) OR (fbss[tiab])) OR (junction failure*[tiab])) OR (recurrent disc*[tiab])) OR (recurrent disk*[tiab]) 635,591

73 recurrent disk*[tiab] 81

72 recurrent disc*[tiab] 489

71 junction failure*[tiab] 58

70 fbss[tiab] 419

69 failed back surgery syndrome*[tiab] 949

68 "failed back surgery syndrome"[mesh] 422

67 adjacent segment disease[tiab] 812

66 spinal cord stimulat*[tiab] 3,833

65 "spinal cord stimulation"[mesh] 1,448

64 post-surgical[tiab] 9,572

63 postsurgical[tiab] 24,018

62 post-operative[tiab] 69,088

61 postoperative[tiab] 581,458

60 persistent spinal pain syndrome[tiab] 14

59 microdiscectom*[tiab] 1,059

58 microdiskectom*[tiab] 92

57 laser surger*[tiab] 3,637

56 laminotom*[tiab] 833

55 laminectom*[tiab] 10,149

54 laminectomy[mesh] 10,216

53 itdd[tiab] 17

52 intrathecal drug delivery[tiab] 386

51 interlaminar implant*[tiab] 1

50 ilif[tiab] 11

49 interlaminar lumbar instrumented fusion*[tiab] 4

48 foraminotom*[tiab] 740

47 foraminotomy[mesh] 218

46 xlif[tiab] 265

45 extreme lateral interbody fusion*[tiab] 207

44 diskectom*[tiab] 785

43 discectom*[tiab] 7,802

42 diskectomy[mesh] 6,423

41 kyphoplast*[tiab] 2,169

40 vertebroplast*[tiab] 3,699

39 cementoplast*[tiab] 248

38 cementoplasty[mesh] 3,183

37 disk arthroplast*[tiab] 61

36 disc arthroplast*[tiab] 767

35 disk replacement*[tiab] 150

34 disc replacement*[tiab] 1,520

33 "total disc replacement"[mesh] 813

32 ((((((((((((((((((((((((((((((tuina[tiab]) OR ("tui na"[tiab])) OR (chuna[tiab])) OR (flexion

distraction[tiab])) OR (cox technique[tiab])) OR (hvla[tiab])) OR (high velocity low amplitude[tiab])) OR (instrument assisted adjust*[tiab])) OR (instrument assisted manipulation*[tiab])) OR (joint manipulation*[tiab])) OR (joint mobilization*[tiab])) OR (joint mobilisation*[tiab])) OR (manipulation under anesthesia[tiab])) OR (manipulation under anaesthesia[tiab])) OR (fibrosis release[tiab])) OR ("musculoskeletal manipulations"[mesh])) OR (musculoskeletal manipulation*[tiab])) OR (manipulative rehabilitation*[tiab])) OR (manipulative therap*[tiab])) OR (manual therap*[tiab])) OR (mobilization therap*[tiab])) OR (mobilisation therap*[tiab])) OR (orthopedic manipulation*[tiab])) OR (orthopaedic manipulation*[tiab])) OR (osteopathic manipulation*[tiab])) OR (chiropractic manipulation*[tiab])) OR (soft tissue mobilization*[tiab])) OR (soft tissue mobilisation*[tiab])) OR (spinal manipulation*[tiab])) OR (spinal mobilization*[tiab])) OR (spinal mobilisation*[tiab]) 22,166

31 spinal mobilisation*[tiab] 18

30 spinal mobilization*[tiab] 120

29 spinal manipulation*[tiab] 1,528

28 soft tissue mobilisation*[tiab] 15

27 soft tissue mobilization*[tiab] 212

26 chiropractic manipulation*[tiab] 458

25 osteopathic manipulation*[tiab] 151

24 orthopaedic manipulation*[tiab] 8

23 orthopedic manipulation*[tiab] 33

22 mobilisation therap*[tiab] 10

21 mobilization therap*[tiab] 121

20 manual therap*[tiab] 3,071

19 manipulative therap*[tiab] 972

18 manipulative rehabilitation*[tiab] 2

17 musculoskeletal manipulation*[tiab] 199

16 "musculoskeletal manipulations"[mesh] 17,866

15 fibrosis release[tiab] 7

14 manipulation under anaesthesia[tiab] 151

13 manipulation under anesthesia[tiab] 392

12 joint mobilisation*[tiab] 63

11 joint mobilization*[tiab] 514

10 joint manipulation*[tiab] 205

9 instrument assisted manipulation*[tiab] 5

8 instrument assisted adjust*[tiab] 2

7 high velocity low amplitude[tiab] 294

6 hvla[tiab] 129

5 cox technique[tiab] 31

4 flexion distraction[tiab] 273

3 chuna[tiab] 49

2 "tui na"[tiab] 36

1 tuina[tiab] 217

**OVID/MEDLINE**

**(1/6/2022)**

Database: Ovid MEDLINE(R) and Epub Ahead of Print, In-Process, In-Data-Review

& Other Non-Indexed Citations and Daily <1946 to January 06, 2022>

Search Strategy:

--------------------------------------------------------------------------------

1 tuina.ti. or tuina.ab. (206)

2 tui na.ti. or tui na.ab. (34)

3 chuna.ti. or chuna.ab. (47)

4 flexion distraction.ti. or flexion distraction.ab. (272)

5 cox technique.ti. or cox technique.ab. (31)

6 hvla.ti. or hvla.ab. (125)

7 high velocity low amplitude.ti. or high velocity low amplitude.ab. (293)

8 instrument assisted adjust*.ti. or instrument assisted adjust*.ab. (0)

9 instrument assisted manipulation*.ti. or instrument assisted manipulation*.ab. (4)

10 joint manipulation*.ti. or joint manipulation*.ab. (201)

11 joint mobilization*.ti. or joint mobilization*.ab. (491)

12 joint mobilisation*.ti. or joint mobilisation*.ab. (62)

13 manipulation under anesthesia.ti. or manipulation under anesthesia.ab. (387)

14 manipulation under anaesthesia.ti. or manipulation under anaesthesia.ab. (148)

15 fibrosis release.ti. or fibrosis release.ab. (7)

16 exp musculoskeletal manipulations/ (17859)

17 musculoskeletal manipulation*.ti. or musculoskeletal manipulation*.ab. (7)

18 manipulative rehabilitation*.ti. or manipulative rehabilitation*.ab. (2)

19 manipulative therap*.ti. or manipulative therap*.ab. (942)

20 manual therap*.ti. or manual therap*.ab. (2696)

21 mobilization therap*.ti. or mobilization therap*.ab. (124)

22 mobilisation therap*.ti. or mobilisation therap*.ab. (11)

23 orthopedic manipulation*.ti. or orthopedic manipulation*.ab. (30)

24 orthopaedic manipulation*.ti. or orthopaedic manipulation*.ab. (8)

25 osteopathic manipulation*.ti. or osteopathic manipulation*.ab. (138)

26 chiropractic manipulation*.ti. or chiropractic manipulation*.ab. (442)

27 soft tissue mobilization*.ti. or soft tissue mobilization*.ab. (205)

28 soft tissue mobilisation*.ti. or soft tissue mobilisation*.ab. (16)

29 spinal manipulation*.ti. or spinal manipulation*.ab. (1429)

30 spinal mobilization*.ti. or spinal mobilization*.ab. (110)

31 spinal mobilisation*.ti. or spinal mobilisation*.ab. (18)

32 1 or 2 or 3 or 4 or 5 or 6 or 7 or 8 or 9 or 10 or 11 or 12 or 13 or 14 or 15 or 16

or 17 or 18 or 19 or 20 or 21 or 22 or 23 or 24 or 25 or 26 or 27 or 28 or 29 or 30 or

31 (21880)

33 exp total disc replacement/ (812)

34 disc replacement*.ti. or disc replacement*.ab. (1444)

35 disk replacement*.ti. or disk replacement*.ab. (146)

36 disc arthroplast*.ti. or disc arthroplast*.ab. (730)

37 disk arthroplast*.ti. or disk arthroplast*.ab. (60)

38 exp cementoplasty/ (3185)

39 cementoplast*.ti. or cementoplast*.ab. (212)

40 vertebroplast*.ti. or vertebroplast*.ab. (3597)

41 kyphoplast*.ti. or kyphoplast*.ab. (2102)

42 exp diskectomy/ (6422)

43 discectom*.ti. or discectom*.ab. (7565)

44 diskectom*.ti. or diskectom*.ab. (706)

45 extreme lateral interbody fusion*.ti. or extreme lateral interbody fusion*.ab.

(183)

46 xlif.ti. or xlif.ab. (198)

47 exp foraminotomy/ (218)

48 foraminotom*.ti. or foraminotom*.ab. (709)

49 interlaminar lumbar instrumented fusion*.ti. or interlaminar lumbar instrumented

fusion*.ab. (4)

50 ilif.ti. or ilif.ab. (10)

51 interlaminar implant*.ti. or interlaminar implant*.ab. (1)

52 intrathecal drug delivery.ti. or intrathecal drug delivery.ab. (358)

53 itdd.ti. or itdd.ab. (17)

54 exp laminectomy/ (10214)

55 laminectom*.ti. or laminectom*.ab. (9895)

56 laminotom*.ti. or laminotom*.ab. (807)

57 laser surger*.ti. or laser surger*.ab. (3480)

58 microdiskectom*.ti. or microdiskectom*.ab. (86)

59 microdiscectom*.ti. or microdiscectom*.ab. (1008)

60 persistent spinal pain syndrome.ti. or persistent spinal pain syndrome.ab. (14)

61 postoperative.ti. or postoperative.ab. (517889)

62 post-operative.ti. or post-operative.ab. (68363)

63 postsurgical.ti. or postsurgical.ab. (15469)

64 post-surgical.ti. or post-surgical.ab. (9461)

65 exp spinal cord stimulation/ (1447)

66 spinal cord stimulat*.ti. or spinal cord stimulat*.ab. (3720)

67 adjacent segment disease.ti. or adjacent segment disease.ab. (730)

68 exp failed back surgery syndrome/ (422)

69 failed back surgery syndrome*.ti. or failed back surgery syndrome*.ab. (859)

70 fbss.ti. or fbss.ab. (407)

71 junction failure*.ti. or junction failure*.ab. (53)

72 recurrent disc*.ti. or recurrent disc*.ab. (495)

73 recurrent disk*.ti. or recurrent disk*.ab. (77)

74 33 or 34 or 35 or 36 or 37 or 38 or 39 or 40 or 41 or 42 or 43 or 44 or 45 or 46

or 47 or 48 or 49 or 50 or 51 or 52 or 53 or 54 or 55 or 56 or 57 or 58 or 59 or 60 or

61 or 62 or 63 or 64 or 65 or 66 or 67 or 68 or 69 or 70 or 71 or 72 or 73 (625945)

75 32 and 74 (**1057**)

**WEB OF SCIENCE**

**(1/7/2022)**

67

#31 AND #66

**418**

66

#32 OR #33 OR #34 OR #35 OR #36 OR #37 OR #38 OR #39 OR #40 OR #41 OR #42 OR #43 OR #44 OR #45 OR #46 OR #47 OR #48 OR #49 OR #50 OR #51 OR #52 OR #53 OR #54 OR #55 OR #56 OR #57 OR #58 OR #59 OR #60 OR #61 OR #62 OR #63 OR #64 OR #65

535,567

65

TS=("recurrent disk*")

93

64

TS=("recurrent disc*")

470

63

TS=("junction failure*")

72

62

TS=(fbss)

679

61

TS=("failed back surgery syndrome*")

935

60

TS=("adjacent segment disease")

915

59

TS=("spinal cord stimulat*")

5,405

58

TS=("post-surgical")

8,855

57

TS=(postsurgical)

15,117

56

TS=("post-operative")

56,898

55

TS=(postoperative)

443,400

54

TS=("persistent spinal pain syndrome")

11

53

TS=(microdiscectom*)

1,403

52

TS=(microdiskectom*)

86

51

TS=("laser surger*")

4,879

50

TS=(laminotom*)

976

49

TS=(laminectom*)

9,231

48

TS=(itdd)

26

47

TS=("intrathecal drug delivery")

452

46

TS=("interlaminar implant*")

2

45

TS=(ilif)

11

44

TS=("interlaminar lumbar instrumented fusion*")

2

43

TS=(foraminotom*)

858

42

TS=(xlif)

344

41

TS=("extreme lateral interbody fusion*")

192

40

TS=(diskectom*)

4,505

39

TS=(discectom*)

7,084

38

TS=(kyphoplast*)

3,075

37

TS=(vertebroplast*)

5,469

36

TS=(cementoplast*)

353

35

TS=("disk arthroplast*")

64

34

TS=("disc arthroplast*")

857

33

TS=("disk replacement*")

199

32

TS=("disc replacement*")

1,686

31

#30 OR #29 OR #28 OR #27 OR #26 OR #25 OR #24 OR #23 OR #22 OR #21 OR #20 OR #19 OR #18 OR #17 OR #16 OR #15 OR #14 OR #13 OR #12 OR #11 OR #10 OR #9 OR #8 OR #7 OR #6 OR #5 OR #4 OR #3 OR #2 OR #1

8,258

30

TS=("spinal mobilisation*")

15

29

TS=("spinal mobilization*")

120

28

TS=("spinal manipulation*")

2,000

27

TS=("soft tissue mobilisation*")

25

26

TS=("soft tissue mobilization*")

222

25

TS=("chiropractic manipulation*")

637

24

TS=("osteopathic manipulation*")

150

23

TS=("orthopaedic manipulation*")

11

22

TS=("orthopedic manipulation*")

30

21

TS=("mobilisation therap*")

11

20

TS=("mobilization therap*")

117

19

TS=("manual therap*")

3,629

18

TS=("manipulative therap*")

1,078

17

TS=("manipulative rehabilitation*")

2

16

TS=("musculoskeletal manipulation*")

271

15

TS=("fibrosis release")

6

14

TS=("manipulation under anaesthesia")

111

13

TS=("manipulation under anesthesia")

331

12

TS=("joint mobilisation*")

71

11

TS=("joint mobilization*")

607

10

TS=("joint manipulation*")

236

9

TS=("instrument assisted manipulation*")

5

8

TS=("instrument assisted adjust*")

0

7

TS=("high velocity low amplitude")

230

6

TS=(hvla)

126

5

TS=("cox technique")

24

4

TS=("flexion distraction")

271

3

TS=(chuna)

68

2

TS=("tui na")

36

1

TS=(tuina)

236

**SCOPUS**

**(1/7/2022)**

68

( ( TITLE-ABS ( tuina ) ) OR ( TITLE-ABS ( "tui na" ) ) OR ( TITLE-ABS ( chuna ) ) OR ( TITLE-ABS ( "flexion distraction" ) ) OR ( TITLE-ABS ( "cox technique" ) ) OR ( TITLE-ABS ( hvla ) ) OR ( TITLE-ABS ( "high velocity low amplitude" ) ) OR ( TITLE-ABS ( "instrument assisted adjust*" ) ) OR ( TITLE-ABS ( "instrument assisted manipulation*" ) ) OR ( TITLE-ABS ( "joint manipulation*" ) ) OR ( TITLE-ABS ( "joint mobilization*" ) ) OR ( TITLE-ABS ( "joint mobilisation*" ) ) OR ( TITLE-ABS ( "manipulation under anesthesia" ) ) OR ( TITLE-ABS ( "manipulation under anaesthesia" ) ) OR ( TITLE-ABS ( "fibrosis release" ) ) OR ( TITLE-ABS ( "musculoskeletal manipulation*" ) ) OR ( TITLE-ABS ( "manipulative rehabilitation*" ) ) OR ( TITLE-ABS ( "manipulative therap*" ) ) OR ( TITLE-ABS ( "manual therap*" ) ) OR ( TITLE-ABS ( "mobilization therap*" ) ) OR ( TITLE-ABS ( "mobilisation therap*" ) ) OR ( TITLE-ABS ( "orthopedic manipulation*" ) ) OR ( TITLE-ABS ( "orthopaedic manipulation*" ) ) OR ( TITLE-ABS ( "osteopathic manipulation*" ) ) OR ( TITLE-ABS ( "chiropractic manipulation*" ) ) OR ( TITLE-ABS ( "soft tissue mobilization*" ) ) OR ( TITLE-ABS ( "soft tissue mobilisation*" ) ) OR ( TITLE-ABS ( "spinal manipulation*" ) ) OR ( TITLE-ABS ( "spinal mobilization*" ) ) OR ( TITLE-ABS ( "spinal mobilisation*" ) ) ) AND ( ( TITLE-ABS ( "failed back surgery syndrome*" ) ) OR ( TITLE-ABS ( fbss ) ) OR ( TITLE-ABS ( "junction failure*" ) ) OR ( TITLE-ABS ( "recurrent disc*" ) ) OR ( TITLE-ABS ( "recurrent disk*" ) ) OR ( ( TITLE-ABS ( "disc replacement*" ) ) OR ( TITLE-ABS ( "disk replacement*" ) ) OR ( TITLE-ABS ( "disc arthroplast*" ) ) OR ( TITLE-ABS ( "disk arthroplast*" ) ) OR ( TITLE-ABS ( cementoplast* ) ) OR ( TITLE-ABS ( vertebroplast* ) ) OR ( TITLE-ABS ( kyphoplast* ) ) OR ( TITLE-ABS ( discectom* ) ) OR ( TITLE-ABS ( diskectom* ) ) OR ( TITLE-ABS ( "extreme lateral interbody fusion*" ) ) OR ( TITLE-ABS ( xlif ) ) OR ( TITLE-ABS ( foraminotom* ) ) OR ( TITLE-ABS ( "interlaminar lumbar instrumented fusion*" ) ) OR ( TITLE-ABS ( ilif ) ) OR ( TITLE-ABS ( "interlaminar implant*" ) ) OR ( TITLE-ABS ( "intrathecal drug delivery" ) ) OR ( TITLE-ABS ( itdd ) ) OR ( TITLE-ABS ( laminectom* ) ) OR ( TITLE-ABS ( laminotom* ) ) OR ( TITLE-ABS ( "laser surger*" ) ) OR ( TITLE-ABS ( microdiscectom* ) ) OR ( TITLE-ABS ( microdiskectom* ) ) OR ( TITLE-ABS ( "persistent spinal pain syndrome" ) ) OR ( TITLE-ABS ( postoperative ) ) OR ( TITLE-ABS ( "post-operative" ) ) OR ( TITLE-ABS ( postsurgical ) ) OR ( TITLE-ABS ( "post-surgical" ) ) OR ( TITLE-ABS ( "spinal cord stimulat*" ) ) OR ( TITLE-ABS ( "adjacent segment disease" ) ) ) ) ...

**489** document results

67

( TITLE-ABS ( "failed back surgery syndrome*" ) ) OR ( TITLE-ABS ( fbss ) ) OR ( TITLE-ABS ( "junction failure*" ) ) OR ( TITLE-ABS ( "recurrent disc*" ) ) OR ( TITLE-ABS ( "recurrent disk*" ) ) OR ( ( TITLE-ABS ( "disc replacement*" ) ) OR ( TITLE-ABS ( "disk replacement*" ) ) OR ( TITLE-ABS ( "disc arthroplast*" ) ) OR ( TITLE-ABS ( "disk arthroplast*" ) ) OR ( TITLE-ABS ( cementoplast* ) ) OR ( TITLE-ABS ( vertebroplast* ) ) OR ( TITLE-ABS ( kyphoplast* ) ) OR ( TITLE-ABS ( discectom* ) ) OR ( TITLE-ABS ( diskectom* ) ) OR ( TITLE-ABS ( "extreme lateral interbody fusion*" ) ) OR ( TITLE-ABS ( xlif ) ) OR ( TITLE-ABS ( foraminotom* ) ) OR ( TITLE-ABS ( "interlaminar lumbar instrumented fusion*" ) ) OR ( TITLE-ABS ( ilif ) ) OR ( TITLE-ABS ( "interlaminar implant*" ) ) OR ( TITLE-ABS ( "intrathecal drug delivery" ) ) OR ( TITLE-ABS ( itdd ) ) OR ( TITLE-ABS ( laminectom* ) ) OR ( TITLE-ABS ( laminotom* ) ) OR ( TITLE-ABS ( "laser surger*" ) ) OR ( TITLE-ABS ( microdiscectom* ) ) OR ( TITLE-ABS ( microdiskectom* ) ) OR ( TITLE-ABS ( "persistent spinal pain syndrome" ) ) OR ( TITLE-ABS ( postoperative ) ) OR ( TITLE-ABS ( "post-operative" ) ) OR ( TITLE-ABS ( postsurgical ) ) OR ( TITLE-ABS ( "post-surgical" ) ) OR ( TITLE-ABS ( "spinal cord stimulat*" ) ) OR ( TITLE-ABS ( "adjacent segment disease" ) ) ) ...

716,072 document results

Set alert Save this search Edit this search Delete this search

66

( TITLE-ABS ( "disc replacement*" ) ) OR ( TITLE-ABS ( "disk replacement*" ) ) OR ( TITLE-ABS ( "disc arthroplast*" ) ) OR ( TITLE-ABS ( "disk arthroplast*" ) ) OR ( TITLE-ABS ( cementoplast* ) ) OR ( TITLE-ABS ( vertebroplast* ) ) OR ( TITLE-ABS ( kyphoplast* ) ) OR ( TITLE-ABS ( discectom* ) ) OR ( TITLE-ABS ( diskectom* ) ) OR ( TITLE-ABS ( "extreme lateral interbody fusion*" ) ) OR ( TITLE-ABS ( xlif ) ) OR ( TITLE-ABS ( foraminotom* ) ) OR ( TITLE-ABS ( "interlaminar lumbar instrumented fusion*" ) ) OR ( TITLE-ABS ( ilif ) ) OR ( TITLE-ABS ( "interlaminar implant*" ) ) OR ( TITLE-ABS ( "intrathecal drug delivery" ) ) OR ( TITLE-ABS ( itdd ) ) OR ( TITLE-ABS ( laminectom* ) ) OR ( TITLE-ABS ( laminotom* ) ) OR ( TITLE-ABS ( "laser surger*" ) ) OR ( TITLE-ABS ( microdiscectom* ) ) OR ( TITLE-ABS ( microdiskectom* ) ) OR ( TITLE-ABS ( "persistent spinal pain syndrome" ) ) OR ( TITLE-ABS ( postoperative ) ) OR ( TITLE-ABS ( "post-operative" ) ) OR ( TITLE-ABS ( postsurgical ) ) OR ( TITLE-ABS ( "post-surgical" ) ) OR ( TITLE-ABS ( "spinal cord stimulat*" ) ) OR ( TITLE-ABS ( "adjacent segment disease" ) ) ...

714,846 document results

Set alert Save this search Edit this search Delete this search

65

TITLE-ABS ( "recurrent disk*" )

95 document results

Set alert Save this search Edit this search Delete this search

64

TITLE-ABS ( "recurrent disc*" )

635 document results

Set alert Save this search Edit this search Delete this search

63

TITLE-ABS ( "junction failure*" )

105 document results

Set alert Save this search Edit this search Delete this search

62

TITLE-ABS ( fbss )

838 document results

Set alert Save this search Edit this search Delete this search

61

TITLE-ABS ( "failed back surgery syndrome*" )

1,022 document results

Set alert Save this search Edit this search Delete this search

60

TITLE-ABS ( "adjacent segment disease" )

794 document results

Set alert Save this search Edit this search Delete this search

59

TITLE-ABS ( "spinal cord stimulat*" )

4,354 document results

Set alert Save this search Edit this search Delete this search

58

TITLE-ABS ( "post-surgical" )

11,429 document results

Set alert Save this search Edit this search Delete this search

57

TITLE-ABS ( postsurgical )

17,333 document results

Set alert Save this search Edit this search Delete this search

56

TITLE-ABS ( "post-operative" )

83,383 document results

Set alert Save this search Edit this search Delete this search

55

TITLE-ABS ( postoperative )

594,603 document results

Set alert Save this search Edit this search Delete this search

54

TITLE-ABS ( "persistent spinal pain syndrome" )

12 document results

Set alert Save this search Edit this search Delete this search

53

TITLE-ABS ( microdiskectom* )

107 document results

Set alert Save this search Edit this search Delete this search

52

TITLE-ABS ( microdiscectom* )

1,112 document results

Set alert Save this search Edit this search Delete this search

51

TITLE-ABS ( "laser surger*" )

4,973 document results

Set alert Save this search Edit this search Delete this search

50

TITLE-ABS ( laminotom* )

841 document results

Set alert Save this search Edit this search Delete this search

49

TITLE-ABS ( laminectom* )

11,106 document results

Set alert Save this search Edit this search Delete this search

48

TITLE-ABS ( itdd )

35 document results

Set alert Save this search Edit this search Delete this search

47

TITLE-ABS ( "intrathecal drug delivery" )

414 document results

Set alert Save this search Edit this search Delete this search

46

TITLE-ABS ( "interlaminar implant*" )

1 document results

Set alert Save this search Edit this search Delete this search

45

TITLE-ABS ( ilif )

20 document results

Set alert Save this search Edit this search Delete this search

44

TITLE-ABS ( "interlaminar lumbar instrumented fusion*" )

5 document results

Set alert Save this search Edit this search Delete this search

43

TITLE-ABS ( foraminotom* )

779 document results

Set alert Save this search Edit this search Delete this search

42

TITLE-ABS ( xlif )

266 document results

Set alert Save this search Edit this search Delete this search

41

TITLE-ABS ( "extreme lateral interbody fusion*" )

186 document results

Set alert Save this search Edit this search Delete this search

40

TITLE-ABS ( diskectom* )

776 document results

Set alert Save this search Edit this search Delete this search

39

TITLE-ABS ( discectom* )

8,326 document results

Set alert Save this search Edit this search Delete this search

38

TITLE-ABS ( kyphoplast* )

2,577 document results

Set alert Save this search Edit this search Delete this search

37

TITLE-ABS ( vertebroplast* )

4,516 document results

Set alert Save this search Edit this search Delete this search

36

TITLE-ABS ( cementoplast* )

257 document results

Set alert Save this search Edit this search Delete this search

35

TITLE-ABS ( "disk arthroplast*" )

87 document results

Set alert Save this search Edit this search Delete this search

34

TITLE-ABS ( "disc arthroplast*" )

815 document results

Set alert Save this search Edit this search Delete this search

33

TITLE-ABS ( "disk replacement*" )

231 document results

Set alert Save this search Edit this search Delete this search

32

TITLE-ABS ( "disc replacement*" )

1,748 document results

Set alert Save this search Edit this search Delete this search

31

( TITLE-ABS ( tuina ) ) OR ( TITLE-ABS ( "tui na" ) ) OR ( TITLE-ABS ( chuna ) ) OR ( TITLE-ABS ( "flexion distraction" ) ) OR ( TITLE-ABS ( "cox technique" ) ) OR ( TITLE-ABS ( hvla ) ) OR ( TITLE-ABS ( "high velocity low amplitude" ) ) OR ( TITLE-ABS ( "instrument assisted adjust*" ) ) OR ( TITLE-ABS ( "instrument assisted manipulation*" ) ) OR ( TITLE-ABS ( "joint manipulation*" ) ) OR ( TITLE-ABS ( "joint mobilization*" ) ) OR ( TITLE-ABS ( "joint mobilisation*" ) ) OR ( TITLE-ABS ( "manipulation under anesthesia" ) ) OR ( TITLE-ABS ( "manipulation under anaesthesia" ) ) OR ( TITLE-ABS ( "fibrosis release" ) ) OR ( TITLE-ABS ( "musculoskeletal manipulation*" ) ) OR ( TITLE-ABS ( "manipulative rehabilitation*" ) ) OR ( TITLE-ABS ( "manipulative therap*" ) ) OR ( TITLE-ABS ( "manual therap*" ) ) OR ( TITLE-ABS ( "mobilization therap*" ) ) OR ( TITLE-ABS ( "mobilisation therap*" ) ) OR ( TITLE-ABS ( "orthopedic manipulation*" ) ) OR ( TITLE-ABS ( "orthopaedic manipulation*" ) ) OR ( TITLE-ABS ( "osteopathic manipulation*" ) ) OR ( TITLE-ABS ( "chiropractic manipulation*" ) ) OR ( TITLE-ABS ( "soft tissue mobilization*" ) ) OR ( TITLE-ABS ( "soft tissue mobilisation*" ) ) OR ( TITLE-ABS ( "spinal manipulation*" ) ) OR ( TITLE-ABS ( "spinal mobilization*" ) ) OR ( TITLE-ABS ( "spinal mobilisation*" ) )

9,425 document results

Set alert Save this search Edit this search Delete this search

30

TITLE-ABS ( "spinal mobilisation*" )

34 document results

Set alert Save this search Edit this search Delete this search

29

TITLE-ABS ( "spinal mobilization*" )

155 document results

Set alert Save this search Edit this search Delete this search

28

TITLE-ABS ( "spinal manipulation*" )

1,633 document results

Set alert Save this search Edit this search Delete this search

27

TITLE-ABS ( "soft tissue mobilisation*" )

27 document results

Set alert Save this search Edit this search Delete this search

26

TITLE-ABS ( "soft tissue mobilization*" )

270 document results

Set alert Save this search Edit this search Delete this search

25

TITLE-ABS ( "chiropractic manipulation*" )

496 document results

Set alert Save this search Edit this search Delete this search

24

TITLE-ABS ( "osteopathic manipulation*" )

199 document results

Set alert Save this search Edit this search Delete this search

23

TITLE-ABS ( "orthopaedic manipulation*" )

46 document results

Set alert Save this search Edit this search Delete this search

22

TITLE-ABS ( "orthopedic manipulation*" )

46 document results

Set alert Save this search Edit this search Delete this search

21

TITLE-ABS ( "mobilisation therap*" )

169 document results

Set alert Save this search Edit this search Delete this search

20

TITLE-ABS ( "mobilization therap*" )

169 document results

Set alert Save this search Edit this search Delete this search

19

TITLE-ABS ( "manual therap*" )

3,686 document results

18

TITLE-ABS ( "manipulative therap*" )

1,111 document results

17

TITLE-ABS ( "manipulative rehabilitation*" )

2 document results

Set alert Save this search Edit this search Delete this search

16

TITLE-ABS ( "musculoskeletal manipulation*" )

11 document results

Set alert Save this search Edit this search Delete this search

15

TITLE-ABS ( "fibrosis release" )

7 document results

Set alert Save this search Edit this search Delete this search

14

TITLE-ABS ( "manipulation under anaesthesia" )

593 document results

Set alert Save this search Edit this search Delete this search

13

TITLE-ABS ( "manipulation under anesthesia" )

593 document results

Set alert Save this search Edit this search Delete this search

12

TITLE-ABS ( "joint mobilisation*" )

90 document results

Set alert Save this search Edit this search Delete this search

11

TITLE-ABS ( "joint mobilization*" )

697 document results

Set alert Save this search Edit this search Delete this search

10

TITLE-ABS ( "joint manipulation*" )

268 document results

Set alert Save this search Edit this search Delete this search

9

TITLE-ABS ( "instrument assisted manipulation*" )

5 document results

Set alert Save this search Edit this search Delete this search

8

TITLE-ABS ( "instrument assisted adjust*" )

0 document results

Set alert Save this search Edit this search Delete this search

7

TITLE-ABS ( "high velocity low amplitude" )

331 document results

Set alert Save this search Edit this search Delete this search

6

TITLE-ABS ( hvla )

156 document results

Set alert Save this search Edit this search Delete this search

5

TITLE-ABS ( "cox technique" )

60 document results

Set alert Save this search Edit this search Delete this search

4

TITLE-ABS ( "flexion distraction" )

315 document results

Set alert Save this search Edit this search Delete this search

3

TITLE-ABS ( chuna )

77 document results

Set alert Save this search Edit this search Delete this search

2

TITLE-ABS ( "tui na" )

51 document results

Set alert Save this search Edit this search Delete this search

1

TITLE-ABS ( tuina )

456 document results

**KOREAMED**

**(1/11/2022)**

68 (tuina[tiab] OR ("tui na"[tiab]) OR (chuna[tiab]) OR ("flexion distraction"[tiab]) OR ("cox

technique"[tiab]) OR (hvla[tiab]) OR ("high velocity low amplitude"[tiab]) OR ("instrument assisted"[tiab]) OR (joint[tiab] AND (manipulation[tiab] OR manipulations[tiab])) OR (joint[tiab] AND (mobilization[tiab] OR mobilizations[tiab])) OR (joint[tiab] AND (mobilisation[tiab] OR mobilisations[tiab])) OR ("manipulation under"[tiab] AND (anesthesia[tiab] OR anaesthesia[tiab])) OR (fibrosis[tiab] AND release[tiab]) OR ("musculoskeletal manipulations"[mh]) OR (musculoskeletal[tiab] AND (manipulation[tiab] OR manipulations[tiab])) OR (manipulative[tiab] AND (rehabiliation[tiab] OR rehabilitations[tiab])) OR (manipulative[tiab] AND (therapy[tiab] OR therapies[tiab])) OR (manual[tiab] AND (therapy[tiab] OR therapies[tiab])) OR (mobilization[tiab] AND (therapy[tiab] OR therapies[tiab])) OR (mobilisation[tiab] AND (therapy[tiab] OR therapies[tiab])) OR (orthopedic[tiab] AND (manipulation[tiab] OR manipulations[tiab])) OR (orthopaedic[tiab] AND (manipulation[tiab] OR manipulations[tiab])) OR (osteopathic[tiab] AND (manipulation[tiab] OR manipulations[tiab])) OR (chiropractic[tiab] AND (manipulation[tiab] OR manipulations[tiab])) OR ("soft tissue"[tiab] AND (mobilization[tiab] OR mobilizations[tiab])) OR ("soft tissue"[tiab] AND (mobilisation[tiab] OR mobilisations[tiab])) OR (spinal[tiab] AND (manipulation[tiab] OR manipulations[tiab])) OR (spinal[tiab] AND (mobilization[tiab] OR mobilizations[tiab])) OR (spinal[tiab] AND (mobilisation[tiab] OR mobilisations[tiab]))) AND ("total disc replacement"[mh] OR ((disc[tiab] OR disk[tiab]) AND replacement[tiab]) OR ((disc[tiab] OR disk[tiab]) AND (arthroplasty[tiab] AND arthroplasties[tiab])) OR (cementoplasty[mh]) OR (cementoplasty[tiab] OR cementoplasties[tiab]) OR (vertebroplasty[tiab] OR vertebroplasties[tiab]) OR (kyphoplasty[tiab] OR kyphoplasties[tiab]) OR (diskectomy[mh]) OR (discectomy[tiab] OR discectomies[tiab]) OR (diskectomy[tiab] OR diskectomies[tiab]) OR ("extreme lateral interbody fusion"[tiab]) OR (xlif[tiab]) OR (foraminotomy[mh]) OR (foraminotomy[tiab] OR foraminotomies[tiab]) OR ("interlaminar lumbar instrumented fusion"[tiab]) OR (ilif[tiab]) OR ("interlaminar implant"[tiab] OR "interlaminar implants"[tiab]) OR ("intrathecal drug delivery"[tiab]) OR (itdd[tiab]) OR (laminectomy[mh]) OR (laminectomy[tiab] OR laminectomies[tiab]) OR (laminotomy[tiab] OR laminotomies[tiab]) OR ("laser surgery"[tiab] OR "laser surgeries"[tiab]) OR (microdiskectomy[tiab] OR microdiskectomies[tiab]) OR (microdiscectomy[tiab] OR microdiscectomies[tiab]) OR ("persistent spinal pain syndrome"[tiab]) OR (postoperative[tiab] OR "post-operative"[tiab]) OR (postsurgical[tiab] OR "post-surgical"[tiab]) OR ("spinal cord stimulation"[mh]) OR ("spinal cord stimulation"[tiab]) OR ("adjacent segment disease"[tiab]) OR ("failed back surgery syndrome"[mh]) OR ("failed back surgery syndrome"[tiab]) OR (fbss[tiab]) OR ("junction failure"[tiab]) OR ("recurrent disc"[tiab] OR "recurrent disk"[tiab])) **75**

67 "total disc replacement"[mh] OR ((disc[tiab] OR disk[tiab]) AND replacement[tiab]) OR

((disc[tiab] OR disk[tiab]) AND (arthroplasty[tiab] AND arthroplasties[tiab])) OR (cementoplasty[mh]) OR (cementoplasty[tiab] OR cementoplasties[tiab]) OR (vertebroplasty[tiab] OR vertebroplasties[tiab]) OR (kyphoplasty[tiab] OR kyphoplasties[tiab]) OR (diskectomy[mh]) OR (discectomy[tiab] OR discectomies[tiab]) OR (diskectomy[tiab] OR diskectomies[tiab]) OR ("extreme lateral interbody fusion"[tiab]) OR (xlif[tiab]) OR (foraminotomy[mh]) OR (foraminotomy[tiab] OR foraminotomies[tiab]) OR ("interlaminar lumbar instrumented fusion"[tiab]) OR (ilif[tiab]) OR ("interlaminar implant"[tiab] OR "interlaminar implants"[tiab]) OR ("intrathecal drug delivery"[tiab]) OR (itdd[tiab]) OR (laminectomy[mh]) OR (laminectomy[tiab] OR laminectomies[tiab]) OR (laminotomy[tiab] OR laminotomies[tiab]) OR ("laser surgery"[tiab] OR "laser surgeries"[tiab]) OR (microdiskectomy[tiab] OR microdiskectomies[tiab]) OR (microdiscectomy[tiab] OR microdiscectomies[tiab]) OR ("persistent spinal pain syndrome"[tiab]) OR (postoperative[tiab] OR "post-operative"[tiab]) OR (postsurgical[tiab] OR "post-surgical"[tiab]) OR ("spinal cord stimulation"[mh]) OR ("spinal cord stimulation"[tiab]) OR ("adjacent segment disease"[tiab]) OR ("failed back surgery syndrome"[mh]) OR ("failed back surgery syndrome"[tiab]) OR (fbss[tiab]) OR ("junction failure"[tiab]) OR ("recurrent disc"[tiab] OR "recurrent disk"[tiab]) 23432

66 "recurrent disc"[tiab] OR "recurrent disk"[tiab] 43

65 "junction failure"[tiab] 1

64 fbss[tiab] 28

63 "failed back surgery syndrome"[tiab] 76

62 "failed back surgery syndrome"[mh] 73

61 "adjacent segment disease"[tiab] 51

60 "spinal cord stimulation"[tiab] 78

59 "spinal cord stimulation"[mh] 77

58 postsurgical[tiab] OR "post-surgical"[tiab] 393

57 postoperative[tiab] OR "post-operative"[tiab] 21676

56 "persistent spinal pain syndrome"[tiab] 0

55 microdiscectomy[tiab] OR microdiscectomies[tiab] 51

54 microdiskectomy[tiab] OR microdiskectomies[tiab] 3

53 "laser surgery"[tiab] OR "laser surgeries"[tiab] 72

52 laminotomy[tiab] OR laminotomies[tiab] 56

51 laminectomy[tiab] OR laminectomies[tiab] 756

50 laminectomy[mh] 754

49 itdd[tiab] 0

48 "intrathecal drug delivery"[tiab] 5

47 "interlaminar implant"[tiab] OR "interlaminar implants"[tiab] 0

46 ilif[tiab] 1

45 "interlaminar lumbar instrumented fusion"[tiab] 1

44 foraminotomy[tiab] OR foraminotomies[tiab] 65

43 foraminotomy[mh] 54

42 xlif[tiab] 4

41 "extreme lateral interbody fusion"[tiab] 7

40 diskectomy[tiab] OR diskectomies[tiab] 18

39 discectomy[tiab] OR discectomies[tiab] 594

38 diskectomy[mh] 549

37 kyphoplasty[tiab] OR kyphoplasties[tiab] 128

36 vertebroplasty[tiab] OR vertebroplasties[tiab] 301

35 cementoplasty[tiab] OR cementoplasties[tiab] 2

34 cementoplasty[mh] 6

33 (disc[tiab] OR disk[tiab]) AND (arthroplasty[tiab] AND arthroplasties[tiab]) 0

32 (disc[tiab] OR disk[tiab]) AND replacement[tiab] 86

31 "total disc replacement"[mh] 48

30 tuina[tiab] OR ("tui na"[tiab]) OR (chuna[tiab]) OR ("flexion distraction"[tiab]) OR ("cox

technique"[tiab]) OR (hvla[tiab]) OR ("high velocity low amplitude"[tiab]) OR ("instrument assisted"[tiab]) OR (joint[tiab] AND (manipulation[tiab] OR manipulations[tiab])) OR (joint[tiab] AND (mobilization[tiab] OR mobilizations[tiab])) OR (joint[tiab] AND (mobilisation[tiab] OR mobilisations[tiab])) OR ("manipulation under"[tiab] AND (anesthesia[tiab] OR anaesthesia[tiab])) OR (fibrosis[tiab] AND release[tiab]) OR ("musculoskeletal manipulations"[mh]) OR (musculoskeletal[tiab] AND (manipulation[tiab] OR manipulations[tiab])) OR (manipulative[tiab] AND (rehabiliation[tiab] OR rehabilitations[tiab])) OR (manipulative[tiab] AND (therapy[tiab] OR therapies[tiab])) OR (manual[tiab] AND (therapy[tiab] OR therapies[tiab])) OR (mobilization[tiab] AND (therapy[tiab] OR therapies[tiab])) OR (mobilisation[tiab] AND (therapy[tiab] OR therapies[tiab])) OR (orthopedic[tiab] AND (manipulation[tiab] OR manipulations[tiab])) OR (orthopaedic[tiab] AND (manipulation[tiab] OR manipulations[tiab])) OR (osteopathic[tiab] AND (manipulation[tiab] OR manipulations[tiab])) OR (chiropractic[tiab] AND (manipulation[tiab] OR manipulations[tiab])) OR ("soft tissue"[tiab] AND (mobilization[tiab] OR mobilizations[tiab])) OR ("soft tissue"[tiab] AND (mobilisation[tiab] OR mobilisations[tiab])) OR (spinal[tiab] AND (manipulation[tiab] OR manipulations[tiab])) OR (spinal[tiab] AND (mobilization[tiab] OR mobilizations[tiab])) OR (spinal[tiab] AND (mobilisation[tiab] OR mobilisations[tiab])) 444

29 spinal[tiab] AND (mobilisation[tiab] OR mobilisations[tiab]) 0

28 spinal[tiab] AND (mobilization[tiab] OR mobilizations[tiab]) 34

27 spinal[tiab] AND (manipulation[tiab] OR manipulations[tiab]) 56

26 "soft tissue"[tiab] AND (mobilisation[tiab] OR mobilisations[tiab]) 0

25 "soft tissue"[tiab] AND (mobilization[tiab] OR mobilizations[tiab]) 32

24 chiropractic[tiab] AND (manipulation[tiab] OR manipulations[tiab]) 8

23 osteopathic[tiab] AND (manipulation[tiab] OR manipulations[tiab]) 0

22 orthopaedic[tiab] AND (manipulation[tiab] OR manipulations[tiab]) 10

21 orthopedic[tiab] AND (manipulation[tiab] OR manipulations[tiab]) 10

20 mobilisation[tiab] AND (therapy[tiab] OR therapies[tiab]) 0

19 mobilization[tiab] AND (therapy[tiab] OR therapies[tiab]) 48

18 manual[tiab] AND (therapy[tiab] OR therapies[tiab]) 123

17 manipulative[tiab] AND (therapy[tiab] OR therapies[tiab]) 5

16 manipulative[tiab] AND (rehabiliation[tiab] OR rehabilitations[tiab]) 0

15 musculoskeletal[tiab] AND (manipulation[tiab] OR manipulations[tiab]) 0

14 "musculoskeletal manipulations"[mh] 12

13 fibrosis[tiab] AND release[tiab] 44

12 "manipulation under"[tiab] AND (anesthesia[tiab] OR anaesthesia[tiab]) 18

11 joint[tiab] AND (mobilisation[tiab] OR mobilisations[tiab]) 1

10 joint[tiab] AND (mobilization[tiab] OR mobilizations[tiab]) 59

9 joint[tiab] AND (manipulation[tiab] OR manipulations[tiab]) 50

8 "instrument assisted"[tiab] 1

7 "high velocity low amplitude"[tiab] 0

6 hvla[tiab] 0

5 "cox technique"[tiab] 0

4 "flexion distraction"[tiab] 2

3 chuna[tiab] 0

2 "tui na"[tiab] 0

1 tuina[tiab] 0

**PEDRO**

**1/12/2022-1/13/2022**

Body Part: lumbar spine, sacro-iliac joint or pelvis

AND

Title/Abstract: tuina 19

"tui na" 1

chuna 3

"flexion distraction" 12

"cox technique" 0

hvla 14

"high velocity low amplitude" 27

"instrument assisted" 0

"joint manipulation" 12

"joint mobilization" 19

"joint mobilisation" 3

"manipulation under anesthesia" 1

"manipulation under anaesthesia" 0

"fibrosis release" 0

"musculoskeletal manipulation" 0

"manipulative rehabilitation" 2

"manipulative therapy" 96

"manual therapy" 179

"mobilization therapy" 2

"mobilisation therapy" 1

"orthopedic manipulation" 0

"orthopaedic manipulation" 1

"osteopathic manipulation" 9

"chiropractic manipulation" 13

"soft tissue mobilization" 4

"soft tissue mobilisation" 0

"spinal manipulation" 177

"spinal mobilization" 20

"spinal mobilisation" 2

Total post-deduplication (manually in EN) **479**

**INDEX TO CHIROPRACTIC LITERATURE**

**1/14/2022**

S1 Article Title:tuina OR Abstract/Notes:tuina 1

S2 Article Title:\"tui na\" OR Abstract/Notes:\"tui na\" 1

S3 Article Title:chuna OR Abstract/Notes:chuna 0

S4 Article Title:\"flexion distraction\" OR Abstract/Notes:\"flexion distraction\" OR

Subject:\"Flexion-Distraction Technique\" 77

S5 Subject:\"Cox Technique\" OR Article Title:\"cox technique\" OR Abstract/Notes:\"cox

technique\" 39

S6 Article Title:hvla OR Abstract/Notes:hvla 68

S7 Article Title:\"high velocity low amplitude\" OR Abstract/Notes:\"high velocity low

amplitude\" 259

S8 Article Title:\"instrument assisted\" OR Abstract/Notes:\"instrument assisted\" 60

S9 Article Title:\"joint manipulation*\" OR Abstract/Notes:\"joint manipulation*\" 51

S10 Article Title:\"joint mobilization*\" OR Abstract/Notes:\"joint mobilization*\" 30

S11 Article Title:\"joint mobilisation*\" OR Abstract/Notes:\"joint mobilisation*\" 1

S12 Article Title:\"joint mobilisation*\" OR Abstract/Notes:\"joint mobilisation*\" 1

S13 Subject:\"Manipulation Under Anesthesia\" OR Article Title:\"manipulation under

anesthesia\" OR Abstract/Notes:\"manipulation under anesthesia\" 40

S14 Subject:\"Manipulation Under Anesthesia\" OR Article Title:\"manipulation under

anesthesia\" OR Abstract/Notes:\"manipulation under anesthesia\" 40

S15 Abstract/Notes:\"manipulation under anaesthesia\" OR Article Title:\"manipulation under

anaesthesia\" 1

S16 Abstract/Notes:\"fibrosis release\" OR Article Title:\"fibrosis release\" 0

S17 Abstract/Notes:\"musculoskeletal manipulation*\" OR Article Title:\"musculoskeletal

manipulation*\" OR Subject:\"Musculoskeletal Manipulations\" 201

S18 Abstract/Notes:\"manipulative rehabilitation\" OR Article Title:\"manipulative

rehabilitation\" 0

S19 Article Title:\"manipulative therap*\" OR Abstract/Notes:\"manipulative therap*\" 0

S20 Article Title:\"manual therap*\" OR Abstract/Notes:\"manual therap*\" 0

S21 Article Title:\"mobilization therap*\" OR Abstract/Notes:\"mobilization therap*\" 0

S22 Article Title:\"mobilisation therap*\" OR Abstract/Notes:\"mobilisation therap*\" 0

S23 Article Title:\"orthopedic manipulation\" OR Abstract/Notes:\"orthopedic manipulation\"

2

S24 Article Title:\"orthopaedic manipulation\" OR Abstract/Notes:\"orthopaedic manipulation\"

0

S25 Article Title:\"osteopathic manipulation\" OR Abstract/Notes:\"osteopathic manipulation\"

10

S26 Article Title:\"chiropractic manipulation\" OR Abstract/Notes:\"chiropractic manipulation\"

231

S27 Article Title:\"soft tissue mobilization\" OR Abstract/Notes:\"soft tissue mobilization\"

44

S28 Article Title:\"soft tissue mobilisation\" OR Abstract/Notes:\"soft tissue mobilisation\"

1

S29 Article Title:\"spinal manipulation*\" OR Abstract/Notes:\"spinal manipulation*\" 856

S30 Article Title:\"spinal mobilization*\" OR Abstract/Notes:\"spinal mobilization*\" 19

S31 Article Title:\"spinal mobilisation*\" OR Abstract/Notes:\"spinal mobilisation*\" 4

S32 Article Title:tuina OR Abstract/Notes:tuina OR Article Title:\"tui na\" OR

Abstract/Notes:\"tui na\" OR Article Title:chuna OR Abstract/Notes:chuna OR Article Title:\"flexion distraction\" OR Abstract/Notes:\"flexion distraction\" OR Subject:\"Flexion-Distraction Technique\" OR Subject:\"Cox Technique\" OR Article Title:\"cox technique\" OR Abstract/Notes:\"cox technique\" OR Article Title:hvla OR Abstract/Notes:hvla OR Article Title:\"high velocity low amplitude\" OR Abstract/Notes:\"high velocity low amplitude\" OR Article Title:\"instrument assisted\" OR Abstract/Notes:\"instrument assisted\" OR Article Title:\"joint manipulation*\" OR Abstract/Notes:\"joint manipulation*\" OR Article Title:\"joint mobilization*\" OR Abstract/Notes:\"joint mobilization*\" OR Article Title:\"joint mobilisation*\" OR Abstract/Notes:\"joint mobilisation*\" OR Article Title:\"joint mobilisation*\" OR Abstract/Notes:\"joint mobilisation*\" OR Subject:\"Manipulation Under Anesthesia\" OR Article Title:\"manipulation under anesthesia\" OR Abstract/Notes:\"manipulation under anesthesia\" OR Subject:\"Manipulation Under Anesthesia\" OR Article Title:\"manipulation under anesthesia\" OR Abstract/Notes:\"manipulation under anesthesia\" OR Abstract/Notes:\"manipulation under anaesthesia\" OR Article Title:\"manipulation under anaesthesia\" OR Abstract/Notes:\"fibrosis release\" OR Article Title:\"fibrosis release\" OR Abstract/Notes:\"musculoskeletal manipulation*\" OR Article Title:\"musculoskeletal manipulation*\" OR Subject:\"Musculoskeletal Manipulations\" OR Abstract/Notes:\"manipulative rehabilitation\" OR Article Title:\"manipulative rehabilitation\" OR Article Title:\"manipulative therap*\" OR Abstract/Notes:\"manipulative therap*\" OR Article Title:\"manual therap*\" OR Abstract/Notes:\"manual therap*\" OR Article Title:\"mobilization therap*\" OR Abstract/Notes:\"mobilization therap*\" OR Article Title:\"mobilisation therap*\" OR Abstract/Notes:\"mobilisation therap*\" OR Article Title:\"orthopedic manipulation\" OR Abstract/Notes:\"orthopedic manipulation\" OR Article Title:\"orthopaedic manipulation\" OR Abstract/Notes:\"orthopaedic manipulation\" OR Article Title:\"osteopathic manipulation\" OR Abstract/Notes:\"osteopathic manipulation\" OR Article Title:\"chiropractic manipulation\" OR Abstract/Notes:\"chiropractic manipulation\" OR Article Title:\"soft tissue mobilization\" OR Abstract/Notes:\"soft tissue mobilization\" OR Article Title:\"soft tissue mobilisation\" OR Abstract/Notes:\"soft tissue mobilisation\" OR Article Title:\"spinal manipulation*\" OR Abstract/Notes:\"spinal manipulation*\" OR Article Title:\"spinal mobilization*\" OR Abstract/Notes:\"spinal mobilization*\" OR Article Title:\"spinal mobilisation*\" OR Abstract/Notes:\"spinal mobilisation*\" 1564 2022-01-14 15:24:09

S33 Article Title:\"disc replacement*\" OR Abstract/Notes:\"disc replacement*\" 5

S34 Article Title:\"disk replacement*\" OR Abstract/Notes:\"disk replacement*\" 0

S35 Article Title:\"disk arthroplast*\" OR Abstract/Notes:\"disk arthroplast*\" 0

S36 Article Title:cementoplast* OR Abstract/Notes:cementoplast* 0

S37 Article Title:vertebroplast* OR Abstract/Notes:vertebroplast* OR \

Subject:\"Vertebroplasty\" 3

S38 Article Title:kyphoplast* OR Abstract/Notes:kyphoplast* 0

S39 Article Title:diskectom* OR Abstract/Notes:diskectom* OR Subject:\"Diskectomy\" 8

S40 Article Title:discectom* OR Abstract/Notes:discectom* 8

S41 Article Title:\"extreme lateral interbody fusion\" OR Abstract/Notes:\"extreme lateral

interbody fusion\" 0

S42 Article Title:xlif OR Abstract/Notes:xlif 0

S43 Article Title:foraminotom* OR Abstract/Notes:foraminotom* 0

S44 Article Title:\"interlaminar lumbar instrumented fusion*\" OR Abstract/Notes:\"interlaminar

lumbar instrumented fusion*\" 0

S45 Article Title:ilif OR Abstract/Notes:ilif 0

S46 Article Title:\"interlaminar implant*\" OR Abstract/Notes:\"interlaminar implant*\" 0

S47 Article Title:\"intrathecal drug delivery\" OR Abstract/Notes:\"intrathecal drug delivery\"

0

S48 Article Title:laminectom* OR Abstract/Notes:laminectom* OR Subject:\"Laminectomy\"

26

S49 Article Title:laminotom* OR Abstract/Notes:laminotom* OR Subject:\"Laminectomy\"

5

S50 Article Title:laminotom* OR Abstract/Notes:laminotom* 0

S51 Article Title:\"laser surger*\" OR Abstract/Notes:\"laser surger*\" 0

S52 Article Title:microdiskectom* OR Abstract/Notes:microdiskectom* 1

S53 Article Title:microdiscectom* OR Abstract/Notes:microdiscectom* 1

S54 Article Title:\"persistent spinal pain syndrome\" OR Abstract/Notes:\"persistent spinal

pain syndrome\" 0

S56 Article Title:postoperative OR Abstract/Notes:postoperative 31

S57 Article Title:\"post-operative\" OR Abstract/Notes:\"post-operative\" 11

S58 Article Title:postsurgical OR Abstract/Notes:postsurgical 34

S59 Article Title:\"post-surgical\" OR Abstract/Notes:\"post-surgical\" 34

S60 Article Title:\"spinal cord stimulat*\" OR Abstract/Notes:\"spinal cord stimulat*\" 0

S61 Article Title:\"adjacent segment disease\" OR Abstract/Notes:\"adjacent segment

disease\" 2

S62 Article Title:\"failed back surgery syndrome\" OR Abstract/Notes:\"failed back surgery

syndrome\" OR Subject:\"Failed Back Surgery Syndrome\" 11

S63 Article Title:fbss OR Abstract/Notes:fbss 0

S64 Article Title:\"junction failure*\" OR Abstract/Notes:\"junction failure*\" 0

S65 Article Title:\"recurrent disc*\" OR Abstract/Notes:\"recurrent disc*\" 0

S66 Article Title:\"recurrent disk*\" OR Abstract/Notes:\"recurrent disk*\" 0

S67 Article Title:\"disc replacement*\" OR Abstract/Notes:\"disc replacement*\" OR Article

Title:\"disk replacement*\" OR Abstract/Notes:\"disk replacement*\" OR Article Title:\"disk

arthroplast*\" OR Abstract/Notes:\"disk arthroplast*\" OR Article Title:cementoplast* OR

Abstract/Notes:cementoplast* OR Article Title:vertebroplast* OR

Abstract/Notes:vertebrooplast* OR Subject:\"Vertebroplasty\" OR Article

Title:kyphoplast* OR Abstract/Notes:kyphoplast* OR Article Title:diskectom* OR

Abstract/Notes:diskectom* OR Subject:\"Diskectomy\" OR Article Title:discectom* OR

Abstract/Notes:discectom* OR Article Title:\"extreme lateral interbody fusion\" OR

Abstract/Notes:\"extreme lateral interbody fusion\" OR Article Title:xlif OR

Abstract/Notes:xlif OR Article Title:foraminotom* OR Abstract/Notes:foraminotom* OR

Article Title:\"interlaminar lumbar instrumented fusion*\" OR Abstract/Notes:\"interlaminar

lumbar instrumented fusion*\" OR Article Title:ilif OR Abstract/Notes:ilif OR Article

Title:\"interlaminar implant*\" OR Abstract/Notes:\"interlaminar implant*\" OR Article

Title:\"intrathecal drug delivery\" OR Abstract/Notes:\"intrathecal drug delivery\" OR

Article Title:laminectom* OR Abstract/Notes:laminectom* OR Subject:\"Laminectomy\"

OR Article Title:laminotom* OR Abstract/Notes:laminotom* OR Subject:\"Laminectomy\"

OR Article Title:laminotom* OR Abstract/Notes:laminotom* OR Article Title:\"laser

surger*\" OR Abstract/Notes:\"laser surger*\" OR Article Title:microdiskectom* OR

Abstract/Notes:microdiskectom* OR Article Title:microdiscectom* OR

Abstract/Notes:microdiscectom* OR Article Title:\"persistent spinal pain syndrome\" OR

Abstract/Notes:\"persistent spinal pain syndrome\" OR Article Title:postoperative OR

Abstract/Notes:postoperative OR Article Title:\"post-operative\" OR

Abstract/Notes:\"post-operative\" OR Article Title:postsurgical OR

Abstract/Notes:postsurgical OR Article Title:\"post-surgical\" OR

Abstract/Notes:\"post-surgical\" OR Article Title:\"spinal cord stimulat*\" OR

Abstract/Notes:\"spinal cord stimulat*\" OR Article Title:\"adjacent segment disease\" OR

Abstract/Notes:\"adjacent segment disease\" OR Article Title:\"failed back surgery

syndrome\" OR Abstract/Notes:\"failed back surgery syndrome\" OR Subject:\"Failed

Back Surgery Syndrome\" OR Article Title:fbss OR Abstract/Notes:fbss OR Article

Title:\"junction failure*\" OR Abstract/Notes:\"junction failure*\" OR Article Title:\"recurrent

disc*\" OR Abstract/Notes:\"recurrent disc*\" OR Article Title:\"recurrent disk*\" OR

Abstract/Notes:\"recurrent disk*\" 142

S68 Article Title:tuina OR Abstract/Notes:tuina OR Article Title:\"tui na\" OR

Abstract/Notes:\"tui na\" OR Article Title:chuna OR Abstract/Notes:chuna OR Article Title:\"flexion distraction\" OR Abstract/Notes:\"flexion distraction\" OR Subject:\"Flexion-Distraction Technique\" OR Subject:\"Cox Technique\" OR Article Title:\"cox technique\" OR Abstract/Notes:\"cox technique\" OR Article Title:hvla OR Abstract/Notes:hvla OR Article Title:\"high velocity low amplitude\" OR Abstract/Notes:\"high velocity low amplitude\" OR Article Title:\"instrument assisted\" OR Abstract/Notes:\"instrument assisted\" OR Article Title:\"joint manipulation*\" OR Abstract/Notes:\"joint manipulation*\" OR Article Title:\"joint mobilization*\" OR Abstract/Notes:\"joint mobilization*\" OR Article Title:\"joint mobilisation*\" OR Abstract/Notes:\"joint mobilisation*\" OR Article Title:\"joint mobilisation*\" OR Abstract/Notes:\"joint mobilisation*\" OR Subject:\"Manipulation Under Anesthesia\" OR Article Title:\"manipulation under anesthesia\" OR Abstract/Notes:\"manipulation under anesthesia\" OR Subject:\"Manipulation Under Anesthesia\" OR Article Title:\"manipulation under anesthesia\" OR Abstract/Notes:\"manipulation under anesthesia\" OR Abstract/Notes:\"manipulation under anaesthesia\" OR Article Title:\"manipulation under anaesthesia\" OR Abstract/Notes:\"fibrosis release\" OR Article Title:\"fibrosis release\" OR Abstract/Notes:\"musculoskeletal manipulation*\" OR Article Title:\"musculoskeletal manipulation*\" OR Subject:\"Musculoskeletal Manipulations\" OR Abstract/Notes:\"manipulative rehabilitation\" OR Article Title:\"manipulative rehabilitation\" OR Article Title:\"manipulative therap*\" OR Abstract/Notes:\"manipulative therap*\" OR Article Title:\"manual therap*\" OR Abstract/Notes:\"manual therap*\" OR Article Title:\"mobilization therap*\" OR Abstract/Notes:\"mobilization therap*\" OR Article Title:\"mobilisation therap*\" OR Abstract/Notes:\"mobilisation therap*\" OR Article Title:\"orthopedic manipulation\" OR Abstract/Notes:\"orthopedic manipulation\" OR Article Title:\"orthopaedic manipulation\" OR Abstract/Notes:\"orthopaedic manipulation\" OR Article Title:\"osteopathic manipulation\" OR Abstract/Notes:\"osteopathic manipulation\" OR Article Title:\"chiropractic manipulation\" OR Abstract/Notes:\"chiropractic manipulation\" OR Article Title:\"soft tissue mobilization\" OR Abstract/Notes:\"soft tissue mobilization\" OR Article Title:\"soft tissue mobilisation\" OR Abstract/Notes:\"soft tissue mobilisation\" OR Article Title:\"spinal manipulation*\" OR Abstract/Notes:\"spinal manipulation*\" OR Article Title:\"spinal mobilization*\" OR Abstract/Notes:\"spinal mobilization*\" OR Article Title:\"spinal mobilisation*\" OR Abstract/Notes:\"spinal mobilisation*\" AND Article Title:\"disc replacement*\" OR Abstract/Notes:\"disc replacement*\" OR Article Title:\"disk replacement*\" OR Abstract/Notes:\"disk replacement*\" OR Article Title:\"disk arthroplast*\" OR Abstract/Notes:\"disk arthroplast*\" OR Article Title:cementoplast* OR Abstract/Notes:cementoplast* OR Article Title:vertebroplast* OR Abstract/Notes:vertebrooplast* OR Subject:\"Vertebroplasty\" OR Article Title:kyphoplast* OR Abstract/Notes:kyphoplast* OR Article Title:diskectom* OR Abstract/Notes:diskectom* OR Subject:\"Diskectomy\" OR Article Title:discectom* OR Abstract/Notes:discectom* OR Article Title:\"extreme lateral interbody fusion\" OR Abstract/Notes:\"extreme lateral interbody fusion\" OR Article Title:xlif OR Abstract/Notes:xlif OR Article Title:foraminotom* OR Abstract/Notes:foraminotom* OR Article Title:\"interlaminar lumbar instrumented fusion*\" OR Abstract/Notes:\"interlaminar lumbar instrumented fusion*\" OR Article Title:ilif OR Abstract/Notes:ilif OR Article Title:\"interlaminar implant*\" OR Abstract/Notes:\"interlaminar implant*\" OR Article Title:\"intrathecal drug delivery\" OR Abstract/Notes:\"intrathecal drug delivery\" OR Article Title:laminectom* OR Abstract/Notes:laminectom* OR Subject:\"Laminectomy\" OR Article Title:laminotom* OR Abstract/Notes:laminotom* OR Subject:\"Laminectomy\" OR Article Title:laminotom* OR Abstract/Notes:laminotom* OR Article Title:\"laser surger*\" OR Abstract/Notes:\"laser surger*\" OR Article Title:microdiskectom* OR Abstract/Notes:microdiskectom* OR Article Title:microdiscectom* OR Abstract/Notes:microdiscectom* OR Article Title:\"persistent spinal pain syndrome\" OR Abstract/Notes:\"persistent spinal pain syndrome\" OR Article Title:postoperative OR Abstract/Notes:postoperative OR Article Title:\"post-operative\" OR Abstract/Notes:\"post-operative\" OR Article Title:postsurgical OR Abstract/Notes:postsurgical OR Article Title:\"post-surgical\" OR Abstract/Notes:\"post-surgical\" OR Article Title:\"spinal cord stimulat*\" OR Abstract/Notes:\"spinal cord stimulat*\" OR Article Title:\"adjacent segment disease\" OR Abstract/Notes:\"adjacent segment disease\" OR Article Title:\"failed back surgery syndrome\" OR Abstract/Notes:\"failed back surgery syndrome\" OR Subject:\"Failed Back Surgery Syndrome\" OR Article Title:fbss OR Abstract/Notes:fbss OR Article Title:\"junction failure*\" OR Abstract/Notes:\"junction failure*\" OR Article Title:\"recurrent disc*\" OR Abstract/Notes:\"recurrent disc*\" OR Article Title:\"recurrent disk*\" OR Abstract/Notes:\"recurrent disk*\" **46**
