# Supplementary material for: Clinician approaches to spinal manipulation for persistent spinal pain after lumbar surgery: systematic review and meta-analysis of individual patient data
Source: Chiropr Man Therap. 2023 Mar 9;31:10. doi: 10.1186/s12998-023-00481-5 (PMC9999664; doi:10.1186/s12998-023-00481-5)
Supplement: Supplementary file 3 — Additional file 3. Sensitivity analysis. [file 12998_2023_481_MOESM3_ESM.docx]

# Supplemental File 3 – Sensitivity Analysis

|  | **Dependent variable** | | | | | | |
| --- | --- | --- | --- | --- | --- | --- | --- |
|  | **Lumbar SMT** | | **Lumbar thrust SMT** | | **Time to SMT < 1 year** | | |
| **Independent variable** | **Odds ratio (95% CI)** | **P-value** | **Odds ratio (95% CI)** | **P-value** | | **Odds ratio (95% CI)** | **P-value** |
| Patient age | 1.05 (0.98-1.12) | .195 | 1.00 (0.96-1.05) | .954 | | 1.03 (0.99-1.07) | .111 |
| Patient sex female (ref. male) | 0.51 (0.09-2.79) | .435 | 1.47 (0.45-4.84) | .529 | | 0.89 (0.29-2.71) | .835 |
| No radiation below gluteal fold (ref. radiation below gluteal fold) | 2.97 (0.17-53.52) | .461 | 3.28 (0.45-23.63) | .239 | | 0.24 (0.03-2.00) | .236 |
| Chiropractor (ref. non chiropractor) | 1.42 (0.24-8.31) | .701 | 32.26 (3.17-327.98) | **.003*** | | 1.88 (0.49-7.27) | .339 |
| No implant (ref. implant) | 1.04 (0.01-75.59) | .985 | 0.27 (0.03-2.20) | .789 | | 0.48 (0.06-4.17) | .508 |
| Motion segments non-reduced (ref. reduced) | 7.60 (0.14-406.79) | .318 | 9.07 (0.97-84.64) | .053 | | 0.36 (0.04-3.34) | .368 |
| Post-surgical imaging (ref. no) | 2.99 (0.34-26.06) | .323 | 4.09 (0.47-35.83) | .203 | | 2.87 (0.66-12.54) | .161 |
| Lumbar SMT (ref. no) | NA | NA | NA | NA | | 2.14 (0.34-13.44) | .419 |
| Time to SMT >1 year (ref. ≤1 year) | 2.73 (0.33-23.01) | .355 | 0.64 (0.15-2.82) | .557 | | NA | NA |
| ***Bold** value indicates P<.05 | | | | | | | |

Table 1: Binary logistic regression sensitivity analysis
